# A tetravalent virus-like particle vaccine designed to display domain III of dengue envelope proteins induces multi-serotype neutralizing antibodies in mice and macaques which confer protection against antibody dependent enhancement in AG129 mice

**DOI:** 10.1371/journal.pntd.0006191

**Published:** 2018-01-08

**Authors:** Viswanathan Ramasamy, Upasana Arora, Rahul Shukla, Ankur Poddar, Rajgokul K. Shanmugam, Laura J. White, Melissa M. Mattocks, Rajendra Raut, Ashiya Perween, Poornima Tyagi, Aravinda M. de Silva, Siddhartha K. Bhaumik, Murali Krishna Kaja, François Villinger, Rafi Ahmed, Robert E. Johnston, Sathyamangalam Swaminathan, Navin Khanna

**Affiliations:** 1 Recombinant Gene Products Group, Molecular Medicine Division, International Centre for Genetic Engineering and Biotechnology, New Delhi, India; 2 Global Vaccines Inc., 801 Capitola Dr., Ste. 11, Durham, NC, United States of America; 3 Department of Microbiology and Immunology, University of North Carolina School of Medicine, Chapel Hill, NC, United States of America; 4 Department of Pediatrics, Division of Infectious Disease, Emory University School of Medicine, Atlanta, GA, United States of America; 5 ICGEB-Emory Vaccine Center, International Centre for Genetic Engineering and Biotechnology, New Delhi, India; 6 Emory Vaccine Center, Emory University School of Medicine, Atlanta, GA, United States of America; 7 Translational Health Science & Technology Institute, NCR Biotech Science Cluster, Faridabad, INDIA; Louisiana State University, UNITED STATES

## Abstract

**Background:**

Dengue is one of the fastest spreading vector-borne diseases, caused by four antigenically distinct dengue viruses (DENVs). Antibodies against DENVs are responsible for both protection as well as pathogenesis. A vaccine that is safe for and efficacious in all people irrespective of their age and domicile is still an unmet need. It is becoming increasingly apparent that vaccine design must eliminate epitopes implicated in the induction of infection-enhancing antibodies.

**Methodology/principal findings:**

We report a *Pichia pastoris*-expressed dengue immunogen, DSV4, based on DENV envelope protein domain III (EDIII), which contains well-characterized serotype-specific and cross-reactive epitopes. In natural infection, <10% of the total neutralizing antibody response is EDIII-directed. Yet, this is a functionally relevant domain which interacts with the host cell surface receptor. DSV4 was designed by in-frame fusion of EDIII of all four DENV serotypes and hepatitis B surface (S) antigen and co-expressed with unfused S antigen to form mosaic virus-like particles (VLPs). These VLPs displayed EDIIIs of all four DENV serotypes based on probing with a battery of serotype-specific anti-EDIII monoclonal antibodies. The DSV4 VLPs were highly immunogenic, inducing potent and durable neutralizing antibodies against all four DENV serotypes encompassing multiple genotypes, in mice and macaques. DSV4-induced murine antibodies suppressed viremia in AG129 mice and conferred protection against lethal DENV-4 virus challenge. Further, neither murine nor macaque anti-DSV4 antibodies promoted mortality or inflammatory cytokine production when passively transferred and tested in an *in vivo* dengue disease enhancement model of AG129 mice.

**Conclusions/significance:**

Directing the immune response to a non-immunodominant but functionally relevant serotype-specific dengue epitope of the four DENV serotypes, displayed on a VLP platform, can help minimize the risk of inducing disease-enhancing antibodies while eliciting effective tetravalent seroconversion. DSV4 has a significant potential to emerge as a safe, efficacious and inexpensive subunit dengue vaccine candidate.

## Author summary

Dengue is mosquito-borne viral disease which is currently a global public health problem. It is caused by four different types of dengue viruses. Nearly a 100 million people a year suffer from overt sickness, which may range from mild fever to potentially fatal disease. A virus-based dengue vaccine was launched for the first time in late 2015. Unexpectedly, this vaccine mimics the dengue viruses in that it appears to elicit disease-enhancing antibodies. To reduce such risk, safer vaccines that eliminate viral proteins responsible for undesirable antibodies are needed. We focused our attention on a small domain of the dengue virus surface protein known as envelope domain III (EDIII). Humans make only a small amount of antibodies against EDIII, but these antibodies are effective in blocking dengue virus from entering cells. We used a yeast expression system to display EDIIIs of all four types of dengue viruses on the surface of non-infectious virus-like particles (VLPs). These VLPs elicited antibodies, in mice and monkeys, which blocked all four dengue virus types and their variants from entering cells in culture. Importantly, these antibodies did not enhance dengue infection in a mouse model.

## Introduction

Dengue is a viral disease spread to humans primarily through mosquito vectors of the genus *Aedes*. The disease is prevalent in >100 tropical and sub-tropical countries of the globe, and is estimated to cause ~390 million infections annually [1]. It is regarded by the World Health Organization (WHO) as one of the fastest spreading vector-borne diseases [2]. Dengue virus (DENV), the etiological agent that causes dengue belongs to the genus *Flavivirus* within the family *Flaviviridae* [3]. There are four antigenically distinct DENV serotypes (DENV-1, DENV-2, DENV-3 and DENV-4), which differ at the amino acid (*aa*) level in the viral envelope by 25–40% [4]. Each DENV serotype is comprised of multiple genotypes, which manifest up to 3% differences at the *aa* level [4, 5].

While in the majority of cases, DENV infection is asymptomatic it can cause symptomatic disease in ~25% cases [1]. This can range from mild dengue fever to severe and potentially fatal dengue hemorrhagic fever and dengue shock syndrome [6]. Severe dengue disease tends to correlate with a heterotypic secondary DENV infection and is held to be the outcome of antibody dependent enhancement (ADE). Antibodies against the primary infecting DENV serotype are unable to neutralize the secondary DENV serotype, but opsonize it and mediate increased infectivity of Fc gamma receptor (FcγR)-bearing monocytes and macrophages to drive up virus production [7]. The ADE hypothesis has provided the rationale for current thinking that a successful dengue vaccine should be capable of inducing strongly neutralizing antibodies (nAbs) to all four DENV serotypes simultaneously [8]. The implicit assumption in this approach that it would circumvent ADE has been belied by phase III trial data [9, 10] and long term safety monitoring of Dengvaxia [11], a live attenuated tetravalent chimeric dengue vaccine. This vaccine was recently approved for use only in adults in regions of high dengue prevalence, where it is projected to bring about a 30% reduction in dengue burden over a thirty year period [12]. Based on the increased hospitalization of younger children, who were found to be seronegative at the time of receiving Dengvaxia, it is now believed that it can simulate a primary DENV infection in seronegative individuals. This could potentially sensitize them to ADE when they encounter a natural DENV infection [13, 14]. It is becoming increasingly evident that the quality of the antibodies elicited by a candidate dengue vaccine is more important than previously realized. This situation warrants the exploration of alternate approaches to dengue vaccine design and development.

The human antibody response to DENV infection tends to be primarily focused towards the generation of weakly-neutralizing and cross-reactive antibodies against the viral structural proteins, pre-membrane (prM) and envelope (E) [15–17]. The prM protein is required for proper folding of the E protein, which is involved in mediating host receptor recognition and fusion of the host and viral membranes [3]. Anti-prM and anti-E antibodies, particularly those that are specific to the fusion-loop (FL) epitope, enhance rather than ablate DENV infection [18–21]. On the other hand, a subset of human DENV-nAbs has been shown to target complex quaternary epitopes discernible only on the mature virion surface [22–25]. Interestingly, some DENV-nAbs also target a carboxy-terminally located domain of the E protein known as envelope domain III (EDIII) [26], which contains multiple dengue complex and serotype-specific neutralizing epitopes [27–29]. Several studies strongly suggest that the host receptor recognition function of the E protein maps to EDIII [30–32], which is discernible on the mature virion surface [33] as well as on recombinant E protein [34, 35]. The accessibility of EDIII on the virion surface is also corroborated by the inclusion of some of its *aa* residues in the footprint of quaternary epitope-recognizing DENV-neutralizing human monoclonal antibodies (mAbs) [23, 25]. Recent work shows that a mAb engineered to bind efficiently to EDIIIs of all four DENVs can abrogate severe dengue disease in a humanized mouse model [36]. Thus, EDIII offers a relatively simple but functional epitope, in comparison to the quaternary epitopes mentioned above. In addition an EDIII-based dengue vaccine holds promise of safety, as it eliminates prM and the rest of the E protein responsible for eliciting antibodies with ADE potential. Interestingly, while the anti-EDIII mAbs have been found to be potent blockers of DENV infectivity [31, 36], they constitute only a minor fraction of the human anti-DENV antibody repertoire [37].

Our work is driven by the hypothesis that an EDIII-based dengue vaccine would not only elicit potent DENV-nAbs but also may help minimize ADE risk. But this would be feasible, if the immunogenicity of EDIII could be augmented. One way to accomplish this would be to display it on the surface of virus-like particles (VLPs). In this context, it is well-documented that the surface antigen (S) of Hepatitis B Virus (HBV), the main component of commercial HBV vaccine, expressed in a recombinant host system, possesses an inherent propensity to self-assemble into VLPs [38–40]. Thus, we designed and created a recombinant Dengue Subunit Virus-like particle tetravalent (DSV4) vaccine candidate based on the HBV S antigen VLP platform, using the methylotrophic yeast *Pichia pastoris* as the expression system. The design of DSV4 was based on two key observations made earlier. In one study, we demonstrated the feasibility of creating an EDIII-based tetravalent antigen by fusing the EDIIIs of the four DENV serotypes ‘in-frame’ *via* flexible peptide linkers [41]. In subsequent work, we showed that HBV S antigen when co-expressed with DENV-2 EDIII fused ‘in-frame’ to the S antigen (in 4:1 ratio), using the *P*. *pastoris* host, self-assembled into stable mosaic DENV-2 EDIII-displaying VLPs, referred to as ES,S_4_ [42]. This approach, similar to that utilized for the RTS, S malaria vaccine [43, 44], provided the rationale for co-expressing 4 copies of HBV S antigen per copy of the EDIII-based tetravalent dengue antigen.

We evaluated the utility of DSV4 as a potential dengue vaccine candidate by investigating: (i) the accessibility and antigenic integrity of EDIII of the four DENV serotypes on DSV4; (ii) the immunogenicity of DSV4 in mice and macaques and its ability to achieve tetravalent seroconversion; and (iii) the protective or enhancing potential of DSV4-induced antibodies in an *in vivo* animal model. Our findings provide proof-of-concept data supporting the utility of DSV4 as a tetravalent dengue vaccine candidate with significant protective efficacy and low enhancement capacity.

## Materials and methods

### Ethics statement

All the experiments using BALB/c mice were conducted at Syngene International Limited, Bangalore (IAEC No. Syngene/IAEC/520/06-2014), in compliance with the guidelines of the Committee for the Purpose of Control and Supervision of Experiments on Animals (CPCSEA) of the Government of India. Experiments using AG129 mice (obtained from Dr. Sujan Shresta, La Jolla Institute for Allergy and Immunology, CA, USA) were performed at Global Vaccines Inc. (GVI), North Carolina, USA, according to GVI’s Institutional Animal Care and Use Committee (IACUC) protocol. AG129 mice experiments were also carried out at ICGEB, New Delhi, in compliance with ICGEB’s Institutional Animal Ethical Committee guidelines and approval. The NHP studies were performed at Emory University. All animals were housed and maintained at the Yerkes National Primate Research Center (YNPRC) of Emory in accordance with the rules and regulations of the Committee on the Care and Use of Laboratory Animal Resources and in accordance with the Weatherall report. Prior to the initiation of the study, the local IACUC approved the entire study including the immunization and collection of samples, which were done under sedation to minimize stress and pain. All the animals were provided water ad libitum, and monkey chow (Purina) supplemented daily with fresh fruit or vegetable for enrichment. The well-being of the animals was monitored daily by animal care and veterinary staff and managed by the Yerkes team. The YNPRC is fully accredited by AAALAC International.

### Cell lines, DENVs, anti-EDIII serotype-specific mAbs and other reagents

Cells, viruses, antibodies and other reagents were obtained from different sources as described in supplementary materials (S1 Appendix). DENV stock titers were determined using a Fluorescence Activated Cell Sorting (FACS) assay [45]. Viral titer was expressed as FACS infectious units (FIU)/ml infected culture supernatant, and was calculated using the formula, FIU/ml = [(% infected cells) x (total number of cells in well) x (dilution factor)]/(volume of virus inoculum) [45].

### DSV4 design and gene construction

DSV4 consists of two proteins, DS and S. The DS protein, encoded by the *DS* gene (GenBank accession no. KM229742), codon-optimized for expression in *P*. *pastoris*, consists of sequences encoding ~104 *aa* each of EDIII-1, -3–4 and -2 (corresponding to DENV-1 West Pac-74, DENV-3 H87/56, DENV-4 H241-P and DENV-2 PR159-S1/69, respectively) and 226 *aa* of S antigen (HBV *adw* serotype), linked in tandem through nucleotide sequences encoding hexa-glycyl linkers, in the order mentioned. The *DS* gene was custom-synthesized and made available in the *P*. *pastoris* vector pPICZa (by DNA2.0 Inc., USA), in which it is flanked on the 5’ and 3’ sides by the *P*. *pastoris AOX1* promoter and transcription terminator, respectively. The *DS* expression cassette was excised from this construct as a *Bgl* II-*Bam* HI fragment and inserted into the *Bam* HI site of 4S-pAO815, a plasmid containing four copies of the S antigen expression cassette [42], to generate the construct DSV4-pAO815 (S1 Fig, panels A-D). This vector was then electroporated into *P*. *pastoris* GS115 strain (Invitrogen, Thermo Fischer Scientific, USA) and transformants selected on plates lacking histidine [42].

### Recombinant DSV4 expression and localization

Yeast cultures were grown at 30°C in buffered glycerol-containing medium (BMGY), and DSV4 expression induced by replacing BMGY with methanol-containing buffered medium, as described earlier [42]. For localization studies, total (T) lysates were prepared and fractionated into membrane-enriched pellet (P) and supernatant (S) fractions. DSV4 in the ‘T’, ‘S’ and urea-solubilized ‘P’ fractions were analyzed by SDS-PAGE followed by silver staining, Western blotting and ELISA using EDIII-specific mAb 24A12 [46] and S antigen-specific mAb 5S as reported earlier [40].

### Purification of recombinant DSV4

DSV4 was purified from induced biomass (~50 g) obtained after 3 days of 2% methanol-induction using a slight modification of our previously described protocol [42] for ES,S_4_ VLPs (supplementary methods, S1 Appendix).

### DSV4 physical characterization

The presence of DS and S proteins in DSV4 was analyzed by SDS-PAGE followed by silver staining. The identity of DS and S proteins was confirmed in immunoblots and ELISAs using EDIII-specific mAb 24A12 [46] and S-specific mAb 5S [40]. The relative ratio of DS and S proteins was determined by densitometric analysis of the Western blot of purified DSV4 developed with mAb 5S. The relative ratio was calculated from the peak area of histogram obtained for DS, S and S dimer using ImageJ software developed by NIH (https://imagej.nih.gov/ij/). Assembly of DSV4 into higher order particulate structures was analyzed by size exclusion chromatography using Superose 6 10/300 GL column (GE Life Sciences, Chicago, USA) and electron microscopy, using uranyl acetate for negative staining. The size distribution profile of DSV4 particles was analyzed by dynamic light scattering (DLS) using Zetasizer Nano Z (Malvern instruments, Malvern, UK).

Antigenic integrity of EDIII epitopes on DSV4 VLPs was assessed using a sandwich ELISA. In this assay DSV4 VLPs were captured using different anti-DENV mAbs and revealed using anti-S antigen mAb-horse radish peroxidase (HRPO) conjugate (from Hepnostika HBsAg Ultra micro ELISA kit). The protocol used was essentially similar to that reported before [42] with slight modifications as described in S1 Appendix.

### Animal immunization

Mouse immunization: For routine immunization, each BALB/c mouse was immunized intramuscularly (i.m.) with 20 μg of DSV4 adsorbed on 500 μg alhydrogel on days 0, 30 and 90. In one early experiment, two additional mouse strains (C57BL-6 and C3H) were also immunized with DSV4 using the same dose and schedule, with the exception that the vaccine was formulated using alhydrogel and monophosphoryl lipid A (MPLA). In most experiments, the number of mice per group was 6. Specific mice numbers are indicated at the appropriate places. As the S: DS ratio in DSV4 VLPs is 4:1, the 20 μg dose, equivalent to 4 μg DS, would provide just under 1 μg of EDIII per DENV serotype. Sera were collected on day 105 for evaluation of antibody titers by ELISA and FACS-based virus-neutralization assay and passive transfer studies. For passive transfer experiments, anti-DENV-2 and anti-DENV-4 antisera were raised in BALB/c mice following multiple immunizations with the cognate live viruses.

Macaque immunization: Macaques in one group (*n* = 6) were immunized (i.m.) with DSV4 (100 μg/macaque) adsorbed on alhydrogel (500 μg) mixed with MPLA (100 μg), on days 0, 28 and 84. The macaque dose was chosen to provide ~4 μg of EDIII per DENV serotype. A second group (*n* = 6) received the same dose of DSV4 formulated in alhydrogel alone without MPLA. Sera were collected on days 42, 84, 98 and 119. Anti-TViDV antiserum for *in vivo* ADE assay (see below) was raised by immunizing macaques twice with a mixture of inactivated DENVs 1–4 (5 μg each) formulated in GVI3000 adjuvant.

### Seroanalysis

Indirect ELISA to detect antibodies directed against each of the five constituents of DSV4 vaccine and FACS-based neutralization test for determining DENV-neutralizing titers in anti-DSV4 antisera were performed using mAb 2H2-Alexa 488 conjugate to identify DENV-infected Vero cells, as described [47]. Serum dilution resulting in 50% reduction in the number of DENV-infected cells with reference to the number of cells infected by DENV in absence of immune serum was defined as the FACS-based Neutralization Titer causing 50% inhibition of DENV infectivity (FNT_50_ titer). *In vitro* ADE assays were performed using K562, as reported [35].

### AG129 mouse challenge experiments

The ADE potential and protective efficacy of DSV4-induced antibodies were tested by either sub-lethal challenge with DENV-2 S221 (5x10^3^ FIU) or lethal challenge with DENV-4 strain 703–4 (10^8^ FIU), respectively, following passive transfer of anti-DSV4-antisera and control sera/mAb into AG129 mice. The sub-lethal (DENV-2 S221) and lethal (DENV-4, 703–4) dosages were pre-determined in early dose-optimization experiments. Viremia was determined on post-challenge day 3 by real time PCR analysis after reverse transcription. In some ADE experiments [48], DENV-2 S221 (2x10^4^ FIU) was partially or fully pre-neutralized with different antibodies/antisera and the resultant immune complexes (ICs) inoculated into AG129 mice (*n* = 9). A subset of these animals (*n* = 3) was euthanized on post-IC inoculation day 3 for the determination of tumor necrosis factor-α (TNF-α) and interleukin-6 (IL-6) in small intestinal tissue homogenates as described (S1 Appendix). Prior monitoring of the kinetics of viremia showed that levels are maximal on post-challenge days 2 and 3. Mortality/morbidity of challenged mice was monitored to generate Kaplan-Meir survival curves as described.

### Statistical analyses

Two-tailed Student *t* test and Mann-Whitney test were used to determine statistical significance of the difference between data sets. Kaplan-Meier survival curves were analyzed by the Log-Rank test for significance. Probability (*p*) levels less than 0.05 were considered as significant. All statistical calculations were performed using GraphPad Prism software (version 7a).

## Results

### DSV4 assembles into VLPs displaying EDIIIs of all four DENV serotypes

**Fig 1:**
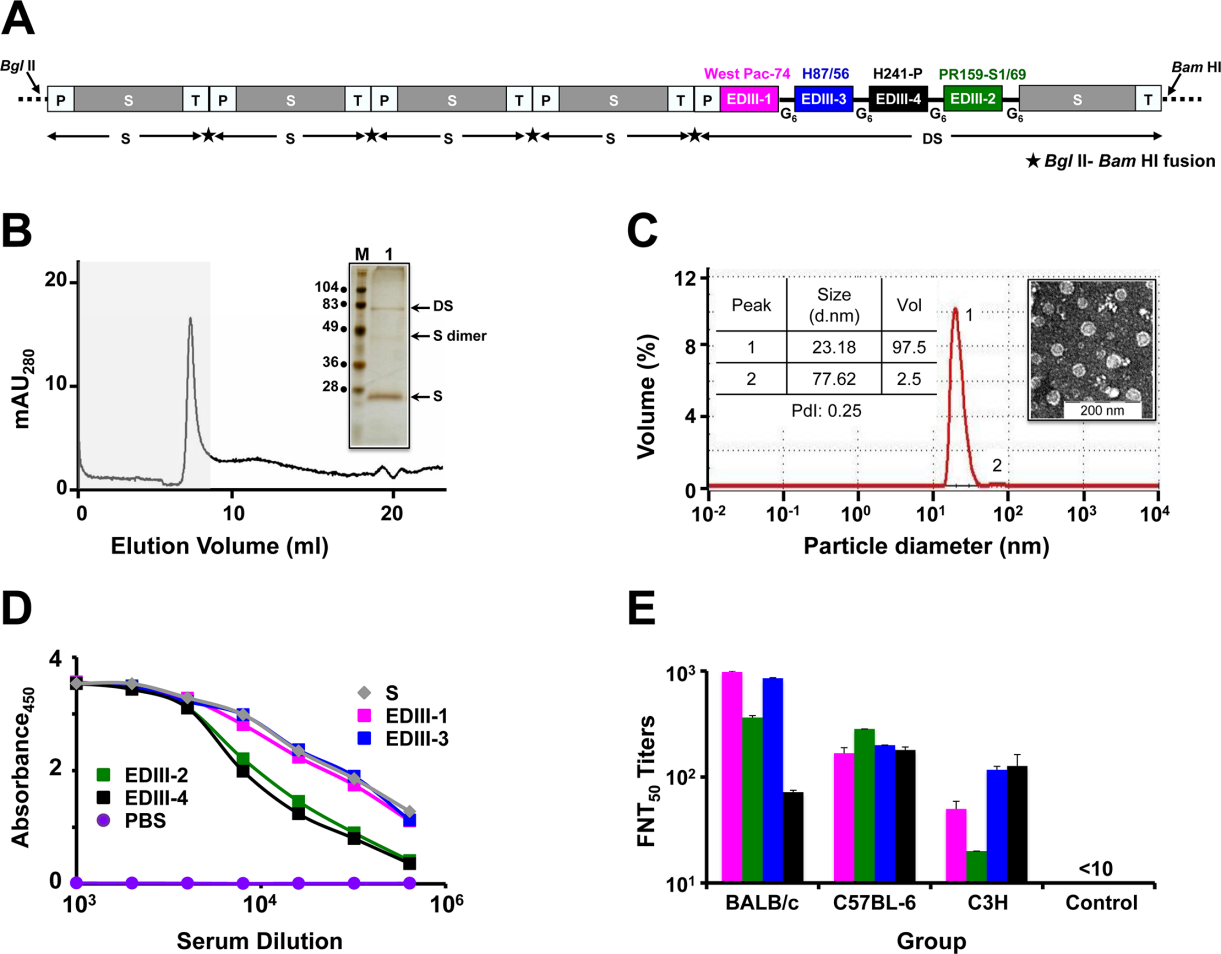
DSV4 assembles into immunogenic VLPs. (A) Schematic representation of DSV4 antigen construct. Magenta, green, blue, and black blocks represent EDIII-encoding domains of DENV-1, -2, -3 and -4, respectively (the specific strains are indicated on the top of respective blocks). EDIIIs were linked to each other and to fusion partner Hepatitis B surface antigen (S; adw serotype; grey colored block), in frame, through hexa-glycyl (G_6_) linkers to encode Dengue-HBsAg (DS) protein. Four expression cassettes of S and one expression cassette of DS were assembled in pAO815 vector between *Bgl* II and *Bam* HI sites. Each expression cassette consists of *5’ AOX1* promoter (P), the recombinant gene (*S* or *DS*) and transcription terminator sequences (T). All the expression cassettes are in tandem, with the black star between adjacent expression cassettes representing *Bgl* II-*Bam* HI fusion site. (B) Elution of purified DSV4 in void volume (grey shaded region) of a Superose 6 column. The inset shows a silver stained gel picture of the material eluted in the void volume (lane ‘1’) with positions of DS, S and dimer of S indicated by arrows on the right. Protein size markers were run in lane ‘M’; their sizes (in kDa) are shown on the left. (C) Volume distribution profile of DSV4 using DLS. The left inset shows DLS parameter values (size, volume and PdI) for peaks 1 and 2. The right inset shows transmission electron microscopic image of DSV4 VLPs. (D) Indirect ELISA reactivity of pooled serum (*n* = 6) collected 15 days after completion of immunization of BALB/c mice (immunized on days 0, 30 and 90) with DSV4 adsorbed on alhydrogel. Serum was analyzed using purified recombinant EDIII-1 (magenta curve), EDIII-2 (green curve), EDIII-3 (blue curve), EDIII-4 (black curve) and HBsAg (grey curve) as capture antigen. PBS-immunized sera (purple curve) served as negative control. Each data point represents the average of duplicates. (E) Groups (*n* = 6) of three different mouse strains (BALB/c, C57BL-6 and C3H) were immunized with DSV4 VLPs as described in methods. A fourth group (*n* = 6, BALB/c) was immunized with S VLPs to serve as negative control. DSV4 and S VLPs were formulated in a mixture of alhydrogel plus MPLA for this experiment alone. Immune sera from all groups were analyzed for nAb titers (FNT_50_ titers) using the FACS assay against WHO reference strains of DENV-1 (magenta bars), DENV-2 (green bars), DENV-3 (blue bars) and DENV-4 (black bars). Mann-Whitney test-derived *p* values for DENV-1 (0.130), DENV-2 (0.959), DENV-3 (0.160) and DENV-4 (0.573) indicated no statistically significant difference in nAb titers between BALB/c and C57BL-6 mice. C3H mice elicited significantly lower nAb titers against DENV-1 (*p* = 0.007) and DENV-2 (*p* = 0.004), compared to BALB/c mice, and against DENV-2 (*p* = 0.006), compared to C57BL-6 mice.

We created DSV4 VLPs by fusing the EDIII-based tetravalent antigen to the HBV S antigen (DS antigen) and co-expressing it with four copies of S antigen in *P*. *pastoris* (Fig 1A, S1 Fig, panels A-D). Co-expressed DS and S proteins in *P*. *pastoris* were associated with the membrane-enriched ‘P’ fraction of methanol-induced lysates (S1 Fig, panels E & F). After optimization of conditions for methanol-induced expression (S1 Fig, panels G & H), DSV4 was purified by hydrophobic interaction chromatography (S2 Fig, panel A) and analyzed by SDS-PAGE and immunoblotting with mAbs specific to its two components (S2 Fig, panel B).

The presence of higher order structures in purified DSV4 was evident based on its elution as a single peak in the void volume when it was subjected to size exclusion chromatography (Fig 1B). SDS-PAGE followed by silver staining of the pooled void volume fractions revealed co-elution of both the protein components of DSV4 (Fig 1B, inset). DLS analysis of this material demonstrated the presence of a predominantly homogenous population (98%, PdI = 0.25) of 20–25 nm sized particles (Fig 1C). Electron microscopic analysis corroborated the presence of discrete VLPs in the purified DSV4 preparation (Fig 1C, inset).

Next we probed the DSV4 VLPs with a panel of DENV-specific conformation-sensitive mAbs [16, 21, 29, 49–55]. One of the key serotype-specific neutralizing epitopes on EDIII is the lateral ridge (LR) epitope [27, 28, 50, 51]. We devised a qualitative sandwich ELISA, wherein we captured the purified VLPs using different type-specific and cross-reactive murine and human DENV E-specific mAbs and revealed the bound VLPs using anti-HBV S mAb-enzyme conjugate. The binding of these mAbs to their specific antigens had been validated in our recent studies [35]. It is to be noted that none of the dengue mAbs used in this experiment recognized HBV S VLPs. Thus, a positive signal is recorded only with DSV4 VLPs which are captured *via* recognition of the EDIII moiety and revealed through recognition of the S moiety. The data on DSV4 reactivity towards these mAbs are summarized in Table 1. DSV4 VLPs showed very good reactivity towards several of these mAbs, most of which were specific to the EDIII-LR epitope. These included type-specific mAbs (which bind to only one serotype), sub-complex specific mAbs (bind to one or more, but not all DENV serotypes), and complex-specific mAbs (bind to all four DENV serotypes). This analysis also revealed the presence of additional EDIII epitopes such as those localized to N-terminal linker, A and B strands, C-C’ loop, D and G strands. Consistent with its design, the DSV4 VLPs failed to be recognized by a battery of fusion loop-recognizing human [16, 52, 53] and murine [49] mAbs as well as by a human anti-prM mAb 2K2 [21]. Collectively, these data led us to the conclusion that the DSV4 VLPs, produced by co-expressing DS and S antigens in *P*. *pastoris*, contain all the five antigenic components, the EDIIIs corresponding to the four serotypes (EDIII-1, EDIII-2, EDIII-3 and EDIII-4) and HBV S, consistent with its design (Fig 1A).

**Table 1:** Antigenic integrity of EDIIIs on DSV4 VLPs using sandwich ELISA.

S.No.^*a*^	Anti-EDIII mAbs ^*b*^	Strongly Neutralizes	Region specificity	ELISA OD	S/Co Ratio^*c*^	Reactivity^*d*^
1	E103	DENV-1	BC loop (LR)	3.96	14.4	++++
2	3H5	DENV-2	A strand, BC loop (LR)	0.77	2.8	+
3	70		A strand, LR	0.48	1.75	+
4	106		A strand, LR	0.51	1.85	+
5	104		C strand/C-C’ loop	0.52	1.89	+
6	8A1	DENV-3	N terminus of A strand, FG loop (LR)	1.30	4.72	++
7	E51		LR	3.90	14.18	++++
8	E88	DENV-4	BC, DE loops (LR)	0.53	1.92	+
9	E76	DENV-2, -4	N terminus of A strand, C-C’ loop, B, D & G strands	0.51	1.85	+
10	E106	DENV-1, -4	A strand, LR	3.60	13.09	++++
11	E113	DENV-1, -2, -4	LR	1.20	4.36	++
12	h-2J20	DENV-1, -3	?	2.50	9.09	+++
13	E61	DENV-1, -2, -3, -4	A and G strands	0.71	2.58	+
14	E77		A strand, BC loop (LR), and G strand	0.81	2.95	+
15	h-2K2	DENV-1, -2, -3, -4	prM	0.06	0.22	-
16	4G2		FL	0.05	0.18	-
17	h-1M7			0.06	0.22	-
18	h-DVC23.3			0.05	0.18	-
19	3H4			0.05	0.18	-
20	h-1N5			0.05	0.18	-

^*a*^S.Nos. 1–14: Anti-EDIII mAbs; S.Nos. 15–20: Non-EDIII mAbs

^*b*^mAbs are described in the following references: h-DVC23.3 [16]; h-2K2 [21]; E103, E106 & E113 [29]; 3H5 & 4G2 [49]; 8A1 [50]; E51, E61, E77 [51]; 3H4 & h-2J20 [52]; h-1M7 & h-1N5 [53]; 70, 104 & 106 [54]; E76 & E88 [55].

^*c*^S/Co: Signal (ELISA OD)/Cut-off; Cut-off = 0.275 (mean ELISA OD obtained using S antigen with each of the 20 mAbs plus 3x SD).

^*d*^Reactivity was graded based on values of *S*/Co:—(<1); + (1.1–3.0); ++ (3.1–5.0); +++(5.1–10); ++++ (>10.1)

?: unknown

The reproducibility of DSV4 purification and its assembly into EDIII-displaying VLPs was ascertained in multiple experiments (S1 Table; S2 Fig, panel C and adjoining table; S3 Fig, panels A-D). The DSV4 VLPs were stable for >1 year in liquid nitrogen and at -80°C, 2 months at 4°C and 1–2 days at 25°C (S3 Fig, panels E & F; S2 Table).

### DSV4 VLPs elicit tetravalent serotype-specific nAbs effective against multiple genotypes in mice

We examined the immunogenicity of DSV4 VLPs in immunocompetent BALB/c mice rather than AG129 mice, despite the latter’s utility as a dengue model, because it is genetically immunocompromised. Sera collected from BALB/c mice immunized with DSV4 VLPs, formulated in alhydrogel adjuvant, were analyzed using an indirect ELISA. In this ELISA, we used all four recombinant EDIII antigens, as well as recombinant S antigen, as the coating antigen, separately, and observed that anti-DSV4 antiserum possessed high titers of antibodies against all its five components (Fig 1D). As we could discern the presence of the potently neutralizing LR epitope of all four DENV serotypes in DSV4 VLPs (Table 1), we wished to analyze the anti-DSV4 antiserum for its ability to neutralize the infectivity of WHO reference DENV strains corresponding to the 4 serotypes, using a FACS based virus-neutralization assay in Vero cells [47]. As we wanted to assess if DSV4 VLPs would be immunogenic in other murine strains as well, we immunized two other strains of mice, C57BL-6 and C3H, apart from BALB/c with DSV4 VLPs (we used MPLA in addition to alhydrogel as adjuvants), and measured nAb titers elicited in all three groups (Fig 1E). This assay showed that DSV4 VLPs could induce nAbs against all four DENV serotypes in all three murine strains. The nAb titers between C57BL-6 and C3H groups were comparable for DENV-1, -3 and -4, with DENV-2 titers being significantly lower in the latter group (*p* = 0.006). The C3H mice elicited significantly lower nAb titers to DENV-1 (*p* = 0.007) and DENV-2 (*p* = 0.004), but not to DENV-3 and DENV-4, compared to BALB/c mice. DENV serotype-wise nAb titers between the BALB/c and C57BL-6 groups were comparable, with overall titers slightly higher in case of the former strain. All further immunogenicity data reported were obtained using the BALB/c strain.

**Fig 2:**
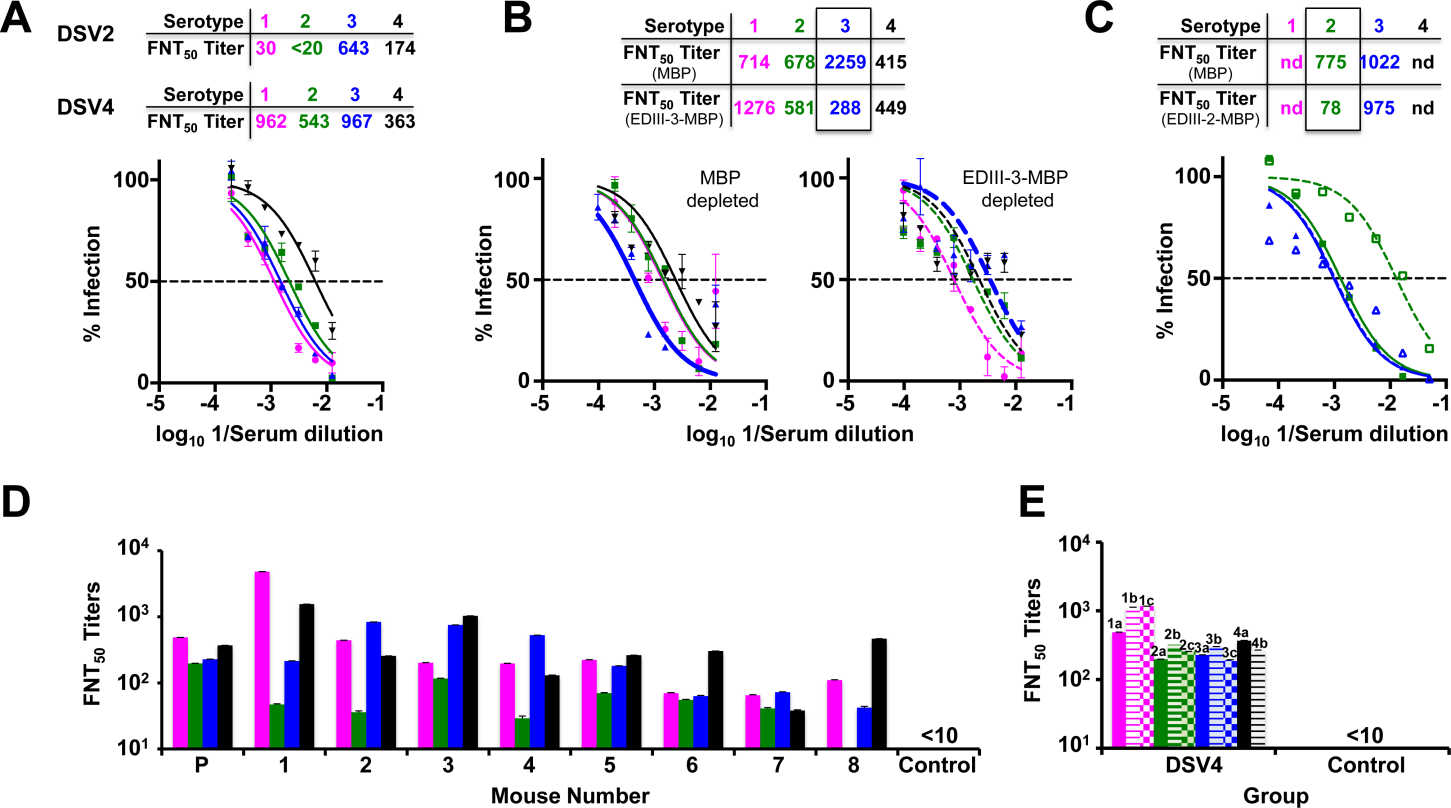
DSV4 elicits tetravalent seroconversion in BALB/c mice. (A) Virus neutralizing activity of anti-DSV4 antiserum (pooled from 6 mice) against WHO reference strains of DENV-1 (magenta), DENV-2 (green), DENV-3 (blue) and DENV-4 (black) as a function of serum dilution, determined using the FACS-based assay. The dashed horizontal line indicates 50% neutralization of virus infectivity. Data points represent mean (*n* = 6) values; the error bars represent SD. Tables above the graph indicate neutralizing antibody titers (FNT_50_) elicited by DSV4 (tetravalent immunogen) and DSV2 (bivalent immunogen). Mann-Whitney test-derived *p* values comparing nAb titers elicited by DSV4 in panel A above (alhydrogel alone as adjuvant) and DSV4 in Fig 1E (alhydrogel + MPLA mixture as adjuvant) against DENV-1 (0.901), -2 (0.710), -3 (0.804) and -4 (0.382) showed no significant differences between the two adjuvant groups. (B) Virus nAb titers against the same four DENV strains indicated in panel A in anti-DSV4 antiserum post-depletion using either MBP (solid curves, left panel) or EDIII-3-MBP (dashed curves, right panel). The four DENV serotypes are indicated using the same colors as in panel A. The table on the top indicates calculated FNT_50_ titers post MBP (middle row) and EDIII-3-MBP (bottom row) depletion. (C). Similar experiment as in panel B except that depletion was with either MBP (solid curves) or EDIII-2-MBP (dashed curves), followed by FNT_50_ titer determination against DENV-2 (green) and DENV-3 (blue). In panels ‘B’ and ‘C’, nAb titers post-depletion with the MBP carrier or specific EDIII-MBP fusion protein are boxed to highlight specific depletion; nd: not done. (D) Determination of FNT_50_ titers against DENV-1, DENV-2, DENV-3 and DENV-4 in individual (1–8) and pooled (P, *n* = 8) antisera from BALB/c mice immunized with DSV4 VLPs. Pooled antisera from a group of BALB/c mice (*n* = 6) immunized with S VLPs served as the control. DENVs used and the colors to indicate the different serotypes are the same as in panel A. (E) Pooled sera from BALB/c mice (*n* = 6 per group) immunized with DSV4 VLPs and control mice, immunized with 1x PBS, were analyzed for FNT_50_ titers against various genotypes corresponding to each of the four DENV serotypes. DENV-1 (magenta) genotypes analyzed were West-Pac 74 (1a), UNC1036 (1b) and UNC1017 (1c); DENV-2 (green) genotypes were S-16803 (2a), IQT2133 (2b) and UNC2037 (2c); DENV-3 (blue) genotypes were CH53489 (3a), UNC3043 (3b) and UNC3001 (3c); and DENV-4 (black) genotypes were TVP360 (4a) and UNC4019 (4b). Data are average of two replicate determinations with error bars shown. Control mice sera did not manifest discernible FNT_50_ titers (<10) even at the lowest serum dilution tested against any of the DENVs.

In contrast to the preceding experiment, we immunized next a fresh group of BALB/c mice with DSV4 VLPs formulated in alhydrogel alone as adjuvant. Antisera from these mice were pooled and tested in DENV neutralization assay against all four serotypes as depicted in Fig 2A. Comparison of the resultant FNT_50_ titers against the four DENV serotypes (Fig 2A, lower Table marked ‘DSV4’) with the corresponding titers obtained in the earlier experiment (Fig 1E) revealed that alhydrogel alone was adequate as an adjuvant for DSV4 in BALB/c mice. Therefore, all further BALB/c immunizations were performed using alhydrogel as the adjuvant.

Are the nAb titers elicited by DSV4 serotype-specific? To address this, we created monovalent VLPs based on the DSV4 platform, in which the four tandem EDIIIs were all of either serotype 3 or serotype 4, and mixed these two together into a bivalent formulation (referred to as DSV2 in Fig 2A, upper table). Antisera from BALB/c mice immunized with DSV2 lacked any significant neutralizing activity towards DENV-1 and DENV-2, but neutralized DENV-3 and DENV-4 almost as efficiently as did the anti-DSV4 antiserum (Fig 2A, lower Table). Thus, the EDIII component elicits nAbs specific to the cognate serotype from which it was derived. To corroborate this, we evaluated the effect of pre-depleting the anti-DSV4 antiserum of anti-EDIII antibodies of a given serotype on nAb titers specific to the cognate serotype. To this end, we pre-incubated anti-DSV4 antiserum with specific recombinant EDIII-MBP fusion proteins immobilized on amylose matrix (S1 Appendix) and then measured the levels of residual DENV-nAb titers (Fig 2B). Pre-depletion of anti-DSV4 antisera on immobilized EDIII-3-MBP led to significant abrogation of nAb titers specific to DENV-3 alone (*p* = 0.028), without appreciable effect on the nAb titers towards the remaining serotypes, as can be seen in Fig 2B (*p* values for DENV-1, -2 and -4 were >0.999, = 0.485 and >0.999, respectively, between the MBP and EDIII-3-MBP depleted antisera, by the Mann-Whitney test). On the other hand, DENV-3 specific nAb titers were essentially unaffected when the pre-depletion was carried out with immobilized EDIII-2-MBP, which specifically decreased nAb titers against DENV-2 (Fig 2C). Collectively, the data leads to the conclusion that DSV4 elicits tetravalent seroconversion and strongly suggests EDIII-focused antibodies elicited by DSV4 are responsible for mostly serotype-specific virus neutralization. Further, we also found that the DSV4 VLP-induced tetravalent immune response in BALB/c mice is durable. In an independent experiment, we could detect significant nAb titers (FNT_50_ titers of 296, 55, 105 and 33, respectively, for DENV-1, -2, -3 and -4) at 3 months after the last immunizing dose. The data on the nAb titers elicited by DSV4 in BALB/c discussed above were obtained using pooled sera. We were interested to know if all mice in a group responded to DSV4 comparably. To this end, we immunized a fresh set of 8 BALB/c mice with DSV4 and analyzed individual sera for FNT_50_ titers against all four DENVs. This experiment revealed that seven of eight mice mounted a tetravalent immune response against all four DENVs with the eighth manifesting trivalent seroconversion (Fig 2D), showing that it is reasonable to use pooled antisera for nAb determinations.

DENVs manifest intra-serotypic variations, known as genotypes. Genotypes within a given serotype can have up to 6% nucleotide sequence divergence in the *E* gene [56]. Could this affect the pan-DENV neutralizing efficacy of anti-DSV4 antibodies? To address this we assessed the virus neutralizing efficacy of antibodies elicited by DSV4 VLPs against multiple genotypes of each of the four DENV serotypes. Fig 2E depicts the DENV-nAb titers elicited by DSV4 VLPs against a total of 11 genotypes, representing the four DENV serotypes. The data show that the polyclonal repertoire of antibodies induced by DSV4 VLPs has adequate breadth of antigenic specificity encompassing multiple genotypes of each of the four DENV serotypes. Control mice immunized with S VLPs (or PBS) did not elicit discernible nAb titers against any of the four WHO strains of DENVs. These data are consistent with our observation that the EDIII sequences of the four specific DENV strains in DSV4 share a high degree of identity with EDIIIs of several hundred DENVs, corresponding to each of the four cognate serotypes in the NCBI database (S3 Table).

### DSV4 VLP-induced antibodies enhance DENV infection in K562 cells but not in AG129 mice

**Fig 3:**
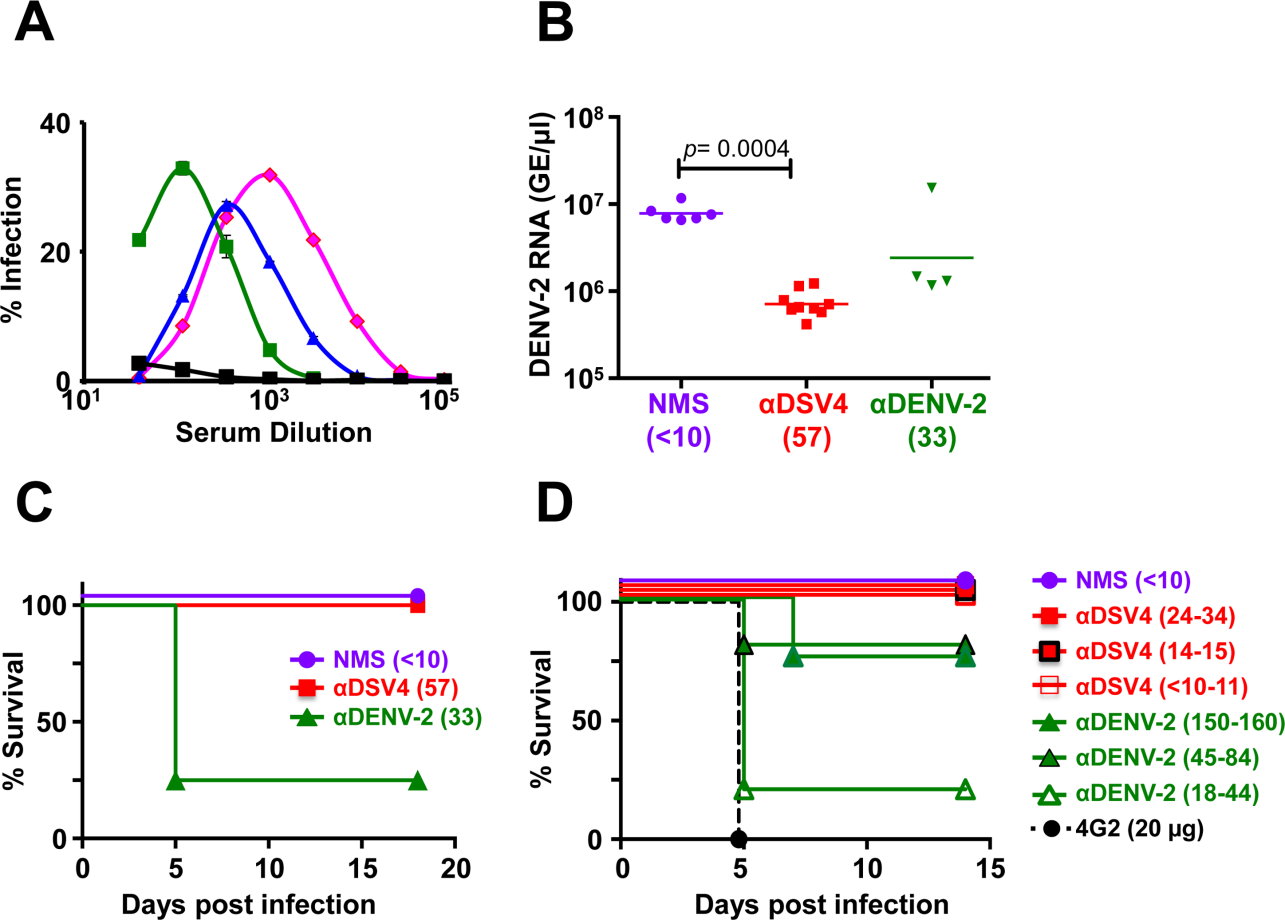
Evaluation of enhancement by antibodies elicited by DSV4 VLPs *in vitro* and *in vivo*. (A) DENV-1 (magenta), DENV-2 (green), DENV-3 (blue) and DENV-4 (black) were pre-incubated with serial three-fold dilutions of anti-DSV4 antiserum followed by FACS determination of infection of K562 cells in culture. (B) AG129 mice received neat NMS (*n* = 6), anti-DSV4 antiserum, αDSV4 (*n* = 9) or anti-DENV-2 antiserum, αDENV-2 (*n* = 4) on day -1. An aliquot of serum was collected from the AG129 mice just before sub-lethal challenge with DENV-2 S221 on day 0 for determination of pre-challenge FNT_50_ titers indicated in parenthesis along the horizontal axis. On day 3, serum viremia was determined by real time analysis. (C) Mice in panel ‘B’ were monitored daily for survival. Pre-challenge FNT_50_ titers are indicated in parenthesis adjacent to the groups. The difference in survival of the NMS and αDSV4 was not significant (*p*>0.999), while those between NMS and αDENV-2 (*p* = 0.016) and αDSV4 and αDENV-2 (*p* = 0.004) groups were significant, based on the Mantel-Cox test. (D) Experimental groups (*n* = 5) were similar to those in panel C, with multiple dilutions of αDSV4 and αDENV-2 antisera (pre-challenge FNT_50_ titers indicated in parenthesis) being used for passive transfer on day -1. A control group received mAb 4G2. All were challenged as before on day 0, and monitored for survival. Survival curves for different groups (panels C and D) at the 100% mark have been slightly displaced to make them visible. The anti-DSV4 antisera used in the *in vitro* and *in vivo* experiments were from different immunizations. The difference in survival of the NMS and αDSV4 was not significant (*p*>0.999), while the survival rates of NMS group (*p* = 0.0002), αDSV4, all dilution groups (*p* = 0.002), αDENV-2, 45–84 group (*p* = 0.014), and αDENV-2, 150–160 group (*p* = 0.004), compared to that of the 4G2 group, were significantly higher, based on the Mantel-Cox test.

Having demonstrated that DSV4 elicits tetravalent seroconversion, we were interested to determine if the anti-DSV4 antibodies could mediate enhancement of DENV infection. We evaluated this *in vitro* by measuring the degree of infection of FcγR-containing K562 cells which are not susceptible to DENV infection in the absence of anti-DENV antibodies. The data shown in Fig 3A reveal that enhancement did occur as evidenced by the characteristic ‘bell-shaped’ curves defining the degree of DENV infection as a function of anti-DSV4 antiserum dilution. Peak enhancement (~30% infection) occurred at antiserum dilutions ranging from ~100 fold (for DENV-2) to ~1000 fold (for DENV-1). The question we addressed next was: would this enhancement seen *in vitro* be evident *in vivo*? To address this, we used the interferon (IFN) α/β and γ receptor-lacking AG129 mouse-based ADE model [57]. Groups of AG129 mice were subjected to passive transfer of normal mouse serum (NMS, from un-immunized BALB/c mice), anti-DSV4 antiserum (αDSV4, from DSV4 VLP-immunized BALB/c mice) or anti-DENV-2 antiserum (αDENV-2, from DENV-2 immunized BALB/c mice), followed by a previously optimized sub-lethal challenge dose of DENV-2 S221 (5x10^3^ FIU/mouse). Experimentally determined pre-challenge DENV-2 nAb titers in the NMS, αDSV4 and the αDENV-2 passive transfer groups were, respectively, <10, 57 and 33 (Fig 3B). Evaluation of DENV genomic RNA levels by real time analysis on day three post-challenge showed relatively higher viremia in the NMS group. Mice in the αDENV-2 group displayed statistically non-significant (*p* = 0.257) reduction in post-challenge viremia. However, anti-DSV4 antiserum could effectively block DENV-2 S221 replication, in the αDSV4 group, as evidenced by a ~1 log reduction in viremia, which was statistically significant (*p* = 0.0004). The day 3 post-challenge time point to assess viremia was chosen based on prior optimization experiments. Interestingly, mice in both NMS- and αDSV4 groups manifested 100% survival while 75% mice in the αDENV-2 group succumbed to the sub-lethal challenge very early (Fig 3C). Given that pre-challenge nAb titer was higher in the αDENV-2 group (33) than in the NMS group (<10), it may be inferred that enhancing antibodies in the anti-DENV-2 antiserum had caused a sub-lethal challenge to become lethal. Interestingly, anti-DSV4 antiserum (pre-challenge nAb titre = 57) not only suppressed viremia significantly compared to NMS, but also did not augment the sub-lethal challenge into a lethal one. This leads to the conclusion that unlike live DENV-2-induced antibodies, the antibodies elicited by DSV4 do not cause ADE *in vivo*. Interestingly, the lack of ADE by anti-DSV4 antibodies *in vivo* (AG129) was not predicted by the observation of ADE *in vitro* (K562).

### Anti-DSV4 antibodies do not possess discernible *in vivo* ADE potential

One possible reason for the inconsistency in outcomes between the *in vitro* and *in vivo* ADE experiments could be that in the former we used serially diluted anti-DSV4 antisera while in the latter we used neat antisera which at best undergo 10–12 fold dilution upon passive transfer into AG129 mice. In other words, would mice in the αDSV4 group experience ADE upon sub-lethal DENV-2 S221 challenge, if the pre-challenge titers were lower? To address this, we used different starting dilutions of passively transferred antiserum to result in a range of lower pre-challenge nAb titers (<10 to 34) in the αDSV4 group. The data are summarized in Fig 3D. As expected, mice which received NMS, survived the sub-lethal challenge. In contrast, all the mice which received 4G2 mAb (at a concentration shown to completely neutralize the sub-lethal challenge virus dose *in vitro*) succumbed on post-challenge day 5 (*p* = 0.0002). This is consistent with the reported behavior of this mAb [48]. On the other hand, it is remarkable that the introduction of anti-DSV4 antiserum at dilutions resulting in the near total lack of pre-challenge DENV-2 nAb titers (<10, corresponding to final dilution of 80-fold) did not predispose AG129 mice to any discernible mortality, compared to the 4G2 group (*p* = 0.0027). In striking contrast, relatively higher levels of pre-challenge DENV-2 nAb titers (18–44, corresponding to a final dilution of 100-fold) resulting from passive transfer of anti-DENV-2 antiserum was associated with very significant mortality (*p* = 0.0014), as seen in the previous experiment. This could largely, but not completely, be counteracted by increasing the pre-challenge DENV-2 nAb titers in the anti-DENV-2 antiserum group to ≥45 (final dilution of 20- to 50-fold). Thus the ADE manifested upon progressive dilution of DENV-2 nAb titers in anti-DENV-2 antiserum (in the range of 20- to 100-fold) is not discernible upon progressive dilution of nAb titers in anti-DSV4 antiserum (in the range of 10- to 80-fold). These data clearly demonstrate the lack of *in vivo* ADE potential of DSV4 VLP-induced antibodies, in contrast to the high ADE potential of DENV-2-induced antibodies and strongly suggest that *in vitro* ADE assay may not reflect *in vivo* ADE potential accurately.

### DENV-2 S221 complexed to anti-DSV4 antibodies does not promote AG129 mortality

**Fig 4:**
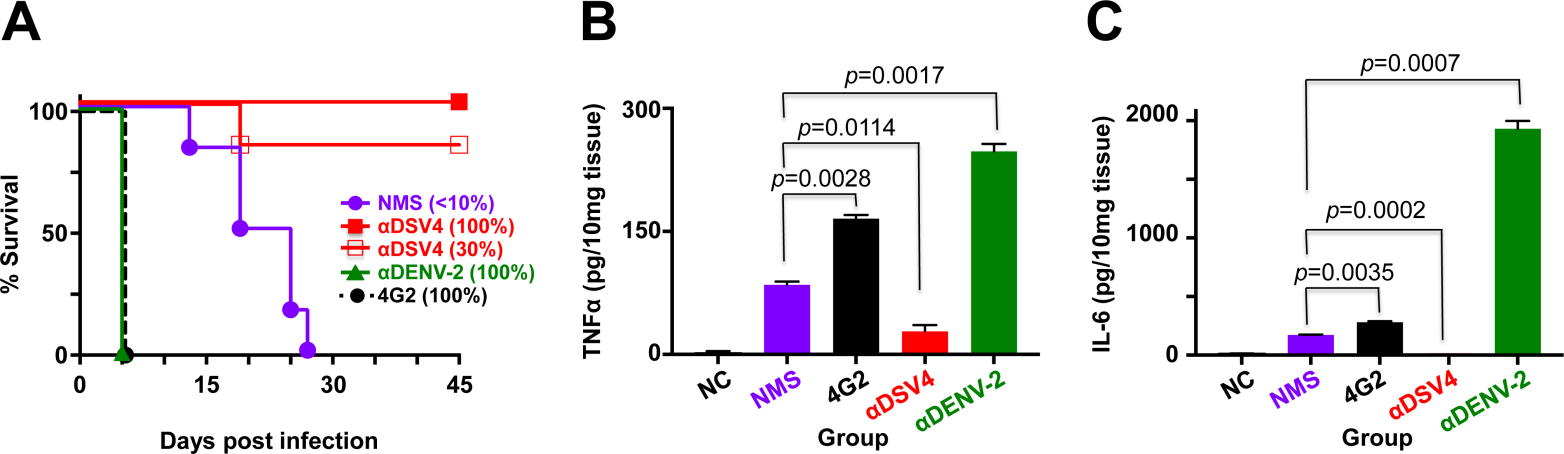
Lack of ADE by anti-DSV4 antibodies correlates with protective efficacy in AG129 mice. (A) Groups (*n* = 6) of AG129 mice were challenged with sub-lethal dose of DENV-2 S221 pre-incubated with normal mouse serum (NMS) or *in vitro*-generated ICs of the same sub-lethal dose of DENV-2 using either sub-neutralizing amounts of anti-DSV4 antiserum [αDSV4 (30%)], or fully neutralizing amounts of anti-DSV4 antiserum [αDSV4 (100%)], anti-DENV-2 antiserum [αDENV-2 (100%)] or mAb 4G2 [4G2 (100%)], and monitored for survival. Survival curves for different groups at the 100% mark have been slightly displaced to make them visible. Survival rates of αDSV4 (30%) IC group (*p* = 0.0009) and αDSV4 (100%) IC group (*p* = 0.0009), compared to that of the 4G2 (100%) group was significant, based on the Log-Rank (Mantel-Cox) test; with respect to the NMS group (challenged with free DENV-2 S221) also, survival was significantly higher for αDSV4 (30%) group (*p* = 0.006) and the αDSV4 (100%) group (*p* = 0.0006). (B) Groups of AG129 mice (*n* = 3) were challenged as described in panel A with the exception that the αDSV4 (30%) IC group was replaced with an untreated group to serve as the negative control (NC). Three days post-inoculation mice were euthanized, small intestines dissected out after perfusion and homogenized. Clarified homogenates were used for the determination of TNF-α using a commercial ELISA kit with purified recombinant murine TNF-α as the reference. (C) The same experiment as in panel B, except that the clarified homogenates were used for IL-6 determination using a commercial ELISA kit with purified recombinant murine IL-6 as the reference. Data shown in panels ‘B’ and ‘C’ are mean values (*n* = 3) with the bars denoting standard deviation. Unpaired *t* test was used to derive the *p* values shown in panels B and C.

The lack of ADE *in vivo* by anti-DSV4 antibodies was corroborated using an alternate strategy [48]. In this approach, we first generated, *in vitro*, completely neutralized immune complexes (ICs) of DENV-2 S221 by incubating a sub-lethal dose with adequate amounts of mAb 4G2, homotypic anti-DENV-2 S221 antiserum or anti-DSV4 antiserum. We also generated partially-neutralized ICs wherein DENV-2 S221 was 30% neutralized, using an appropriate dilution of anti-DSV4 antiserum [αDSV4 (30%)]. This was done to simulate lower nAb concentrations implicated in causing ADE. All four different kinds of ICs were injected separately into groups (*n* = 6) of AG129 mice and compared with a fifth group (control) which received an equivalent dose of free DENV-2 S221 (not complexed to any anti-dengue antibody, pre-incubated with NMS). Unlike in the passive transfer experiments, we monitored these mice for a longer duration (up to 45 days) to examine if the lack of enhancement seen above with anti-DSV4 antibodies (Fig 3, panels C & D) would be overcome by increased DENV-2 S221 replication at later times. This experiment was done twice and the data from one of these are depicted in Fig 4A. Consistent with conclusions from the data in Fig 3D, as well as earlier reported studies [48], injection of the mAb 4G2-ICs resulted in 100% mortality in 5 days. In contrast, αDSV4 ICs did not result in any mortality. Remarkably, the partially neutralized ICs [αDSV4 (30%)] also did not result in any significant mortality for the entire duration of the experiment. The survival of control group which received free DENV-2 S221 (pre-incubated with NMS) began to decline after ~2 weeks, with all mice succumbing by ~4 weeks, despite homotypic FNT_50_ titers of ~3,000–4,000 in the preceding 24–48 hours. Clearly, these high nAb titers elicited by the virus could not preclude ADE. Consistent with this notion, the mortality of the group administered with fully neutralized ICs made using homotypic anti-DENV-2 antiserum (αDENV-2 group) was high and equivalent to those that received mAb 4G2-ICs. In both these instances, the virtual lack of any discernible protection against sub-lethal challenge, despite the DENV-2 S221 in the ICs being 100% neutralized by mAb 4G2 and the homotypic anti-DENV-2 antiserum, reflects the enormous ADE potential of these antibodies. In contrast, such ADE is not evident in case of the ICs generated using anti-DSV4 antibodies, even at sub-neutralizing concentrations, typified by the αDSV4 (30%) group.

It has been documented that ADE in AG129 mice caused by ICs made of mAb 4G2 and DENV-2 S221 is associated with increased vascular permeability and the production of TNF-α and IL-6, especially in the small intestines [48]. We examined these two pro-inflammatory cytokine markers of small intestinal pathology in the various IC-inoculation groups (*n* = 3, set up in parallel) described in the previous experiment. Data on the levels of these two cytokines in the small intestines of AG129 mice, three days after IC inoculation are depicted in Fig 4 (panels B and C). Both TNF-α and IL-6 levels were elevated upon DENV-2 S221 challenge (NMS group) compared to un-challenged controls (NC group). This was further elevated in mice which received the mAb 4G2-containing ICs. A remarkable spurt in the levels of these cytokines was evident in the small intestines of mice inoculated with ICs generated using anti-DENV-2 S221 antisera instead (αDENV-2 group). The mice in these two latter groups succumbed by post-IC inoculation day 5. In striking contrast, TNF-α and IL-6 levels in the small intestines of mice in the αDSV4 IC group were reduced below those in the NMS group (challenged with free DENV-2 S221). It is noteworthy that these mice did not display any mortality (Fig 4A). Collectively, the early cytokine data are quite consistent with the survival data in the *in vivo* ADE experiments. Taken together, these findings suggested that the antibodies elicited by DSV4 VLPs are qualitatively superior in the context of affording a prolonged survival advantage to sub-lethal DENV-2 challenge in these experiments. The data thus far provide evidence that antibodies elicited by DSV4 not only do not mediate ADE, but also apparently confer protection. These data suggest that it is not just nAb titers, but nAbs devoid of enhancing potential, which offer a better correlate of protection.

### Anti-DSV4 antibodies afford significant protection against lethal DENV-4 challenge in AG129 mice

**Fig 5:**
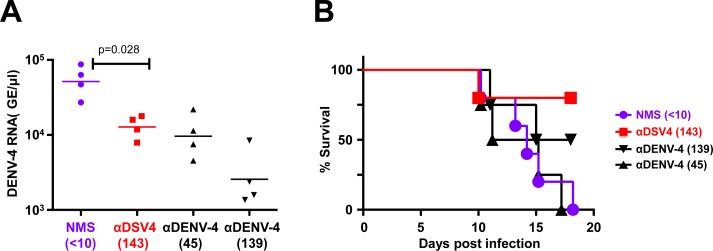
Passively transferred anti-DSV4 BALB/c antiserum suppresses viremia and confers protection to AG129 mice against lethal DENV-4 challenge. (A) Groups (*n* = 4) of AG129 mice received NMS, anti-DSV4 antiserum (α-DSV4), or anti-DENV-4 antiserum (αDENV-4) at 2 dilutions (to result in two different pre-challenge nAb titers) on day -1. Pre-challenge FNT_50_ titers are shown in parenthesis below. On day 0 the mice were challenged with a lethal dose of DENV-4 703–4 virus. Serum viremia was determined on day 3. The difference in viremia between the NMS and αDSV4 groups (*p* = 0.028), NMS and αDENV-4, 1:3 dilution group (*p* = 0.028), and αDENV-4, 1:10 dilution group (*p* = 0.028) were significant (Mann-Whitney test). (B) Mice in ‘A’ were monitored for survival. The difference in survival between NMS and αDSV4 groups (*p* = 0.027), αDSV4 and αDENV-4, 1:10 dilution group (p = 0.036) were significant (Mantel-Cox test).

The experiments described above demonstrated that passively transferred anti-DSV4 antibodies suppressed DENV-2 viremia and a sub-lethal DENV-2 infection from turning lethal in AG129 mice (Fig 3, panels B-D and Fig 4A). While these data indicate protection against ADE, they do not constitute evidence of protection against a lethal infection. DSV4 VLPs tended to elicit relatively lower nAb titers against DENV-4, compared to the remaining serotypes, in BALB/c mice (Figs 1E and 2A). Would such titers be adequate to protect against lethal DENV-4 challenge? To address this, we used DENV-4, 703–4, a recently reported challenge DENV strain [58]. We performed an initial dose escalation study to determine the lethal dose of DENV-4, 703–4. As done earlier, immune serum from DSV4 VLP-immunized BALB/c mice was passively transferred into AG129 mice (αDSV4 group, pre-challenge DENV-4 FNT_50_ = 143), which were then challenged with a lethal dose of DENV-4, strain 703–4. For comparison, we also challenged groups of AG129 mice which had received either NMS or anti-DENV-4 antiserum (αDENV-4 groups, at two different dilutions, 1:3 and 1:10) through passive transfer. On day 3 post-challenge, viremia was evaluated by real time analysis of DENV-4 genomic RNA (Fig 5A). The data showed that the αDSV4 group manifested statistically significant reduction in DENV-4 viremia compared to the NMS control group (*p* = 0.028). A comparable reduction in viremia was also evident in the group passively transferred with ten-fold diluted anti-DENV-4 antiserum (pre-challenge DENV-4 FNT_50_ = 45). This was suppressed even further in the group that received three-fold diluted anti-DENV-4 antiserum (pre-challenge DENV-4 FNT_50_ = 139). Despite this, mortality in these two latter groups was 100% and 50%, respectively (Fig 5B). Remarkably, survival rate was 75% for the mice that received anti-DSV4 antiserum (*p* = 0.027). It is noteworthy that the αDSV4 group and the αDENV-4 1:3 group had similar pre-challenge titers (FNT_50_ ~140), with viremia being lower in the latter compared to the former group. However, this lowered viremia was statistically non-significant (*p* = 0.057). Clearly, high nAb titers and lower viremia in the αDENV-4 1:3 group did not correlate with better survival against lethal challenge. Once again, the data from these experiments strengthen the notion that antibodies elicited by DSV4 VLPs are qualitatively superior, in terms of protective efficacy, compared to antibodies elicited by live DENV-4 infection.

### Macaque-induced anti-DSV4 nAbs manifest negligible ADE potential in the AG129 mouse model

**Fig 6:**
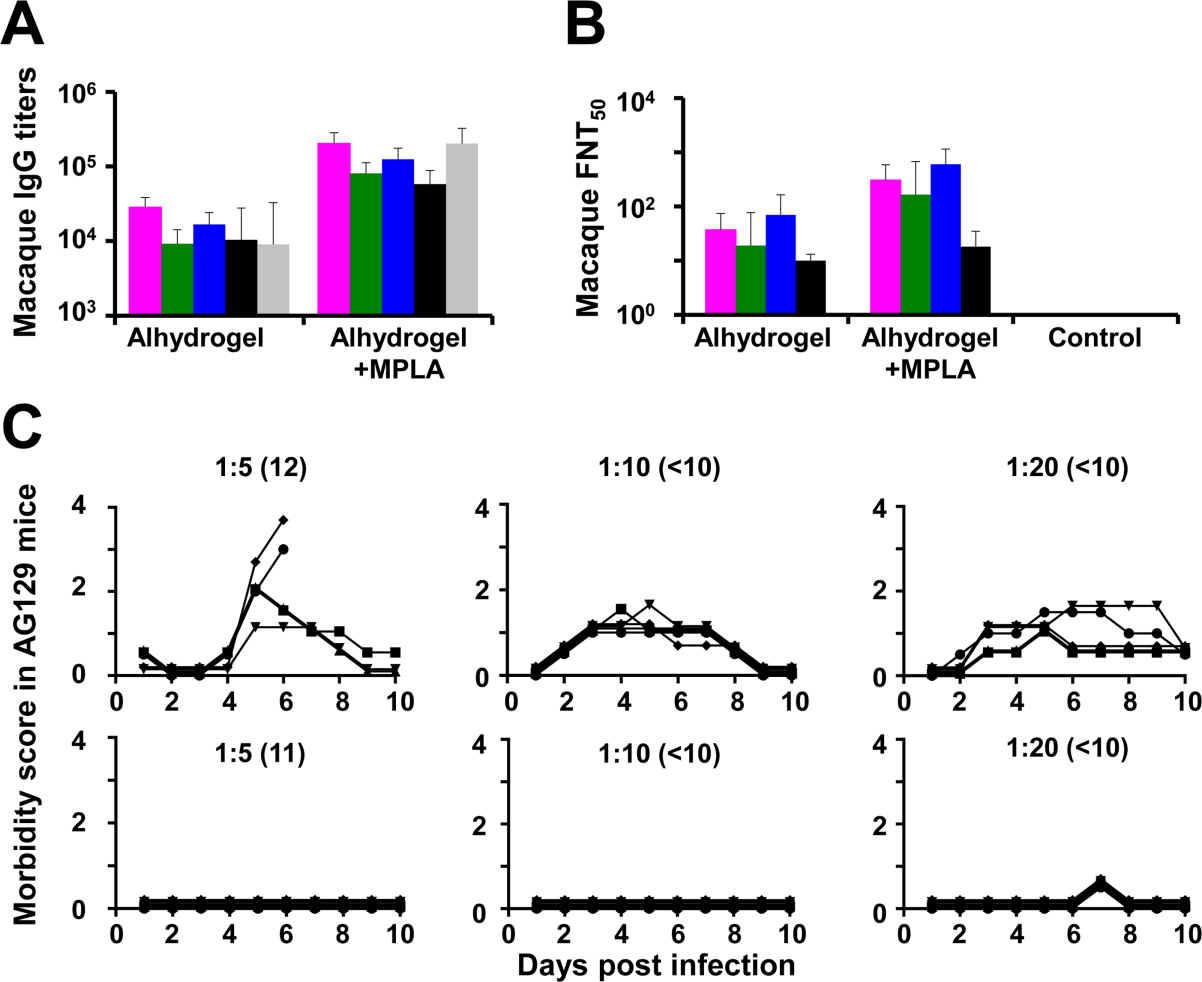
Evaluation of DSV4 in macaques. (A) Serum IgG titers were determined in macaques (*n* = 6) which had been immunized with DSV4 formulated either in alhydrogel alone (Alhydrogel) or in alhydrogel with MPLA (Alhydrogel+MPLA) by indirect ELISA, using purified recombinant EDIII-1 (magenta), EDIII-2 (green), EDIII-3 (blue), EDIII-4 (black) or S (grey) protein as the coating antigen. ELISA titers (GMTs) obtained using the double adjuvant formulation, were significantly higher than those obtained using the single adjuvant formulation, for each of the five coating antigens. Differences in ELISA GMTs between the two adjuvant groups for EDIII-1, EDIII-2, EDIII-3, EDIII-4 and S coating antigens were significant, with *p* values of 0.002, 0.002, 0.002, 0.008 and 0.002, respectively, using the Mann-Whitney test. (B) Neutralizing antibody titers (FNT_50_) against WHO reference strains of DENV-1 (magenta), DENV-2 (green), DENV-3 (blue) and DENV-4 (black) in macaque immune sera from the two adjuvant groups in panel A were determined using the FACS-based assay. Differences in neutralizing GMTs between the two adjuvant groups were significant for DENV-1 (*p* = 0.004), DENV-2 (*p* = 0.015) and DENV-3 (*p* = 0.002), but not DENV-4 (*p* = 0.225), by the Mann-Whitney test. In both panels ‘A’ and ‘B’, data shown are geometric mean values (*n* = 6); error bars denote SD. (C) ADE potential of macaque immune sera was analyzed in AG129 mice. Data show the morbidity scores of AG129 mice (*n* = 5; symbols denote individual mice, monitored over 10 days) passively transferred with pooled macaque anti-TviDV antiserum (top panels) or macaque anti-DSV4 antiserum (bottom panels) at three dilutions, as indicated and challenged a day later with a sub-lethal dose of DENV-2 S221. Pre-challenge FNT_50_ titres are indicated in parenthesis adjacent to the corresponding dilutions used. Morbidity scores were as follows: 0.5, mild ruffled fur; 1, ruffled fur; 1.5, loose stools, eyes compromised; 2, lethargy; 2.5, limited mobility from stimulation, hunching; 3, Not moving, or >20% initial weight loss; 4, moribund. Mice were euthanized when score was 3 or higher.

We also evaluated the immunogenicity of DSV4 VLPs in macaques, using two adjuvants: alhydrogel and alhydrogel + MPLA. Individual sera were analyzed by indirect ELISA using the recombinant monovalent EDIII proteins, corresponding to the four DENV serotypes, as well as recombinant HBV S antigen, as the coating antigens (S4 Fig, panel A). Fig 6A depicts the ELISA results (as geometric mean titers, GMT), using macaque sera drawn at 2 weeks after the last immunization. In contrast to mice, we observed that the dual adjuvant formulation mediated an overall augmentation of the immunogenicity of DSV4, compared to the alhydrogel only formulation. The IgG titers against each of the five coating antigens were approximately an order of magnitude higher when immunization was with DSV4 formulated in alhydrogel + MPLA as compared to that formulated in alhydrogel alone. This was reflected in the DENV nAb titers as well, shown in Fig 6B. As seen with control murine antisera, control macaque antisera obtained from PBS-immunized animals did not manifest any detectable DENV-specific nAb titers. Determination of nAb titers in pooled macaque anti-DSV4 antisera following depletion of EDIII-specific antibodies revealed varying degrees of cross-reactivity among the DENV serotypes (S4 Table). Analysis of nAb titers in individual macaques revealed that while 5 of 6 macaques manifested seroconversion to at least three serotypes when immunized with DSV4 formulated in alhydrogel, all 6 macaques immunized with DSV4 in alhydrogel + MPLA seroconverted against at least three serotypes (S4 Fig, panel B).

Finally, we also examined the potential of the macaque-raised anti-DSV4 antiserum to enhance infection in the AG129/sub-lethal DENV-2 S221 challenge model. For comparison, we also performed passive transfer with an antiserum raised in macaques immunized with an ‘in house’ tetravalent mixture of inactivated DENVs (TviDV). Each of these two macaque antisera pools was passively transferred into AG129 mice at three different dilutions as indicated (Fig 6C). At 24 hours post-passive transfer, the mice were challenged with the sub-lethal dose of DENV-2 S221. The TviDV antiserum, at all three dilutions tested, sensitized the AG129 mice to DENV-2 S221 challenge, based on several indicators of morbidity (Fig 6C, upper three panels). In striking contrast, anti-DSV4 antiserum did not sensitize the AG129 mice to viral challenge at all three dilutions (Fig 6C, lower three panels). It is interesting to note that at comparable pre-challenge DENV-2 nAb titers anti-DSV4 antiserum and anti-TviDV antiserum manifested contrasting outcomes in this experiment, corroborating the qualitative superiority of antibodies in the former compared to the latter antiserum. The observed lack of discernible ADE by macaque-raised anti-DSV4 antibodies, in the AG129 model, is consistent with the lack of infection enhancement by BALB/c-raised anti-DSV4 antibodies, in the same ADE model, and lends strength to the conclusion that DSV4 is not associated with the induction of disease-enhancing antibodies. This is a vital and desirable feature of the DSV4 VLP candidate as it can potentially circumvent or minimize the possibility of ADE in vaccine recipients.

## Discussion

The search for an ideal dengue vaccine is far from over as the recently launched live attenuated dengue vaccine appears to be linked to possible ADE in young children. To make a dengue vaccine safe it is necessary to eliminate or minimize the risk of potential ADE by avoiding epitopes implicated in the induction of enhancing antibodies. Several recent studies have led to the recognition that DENVs subvert a major part of the human antibody response towards generating weakly neutralizing, cross-reactive enhancing antibodies [15–17]. This should no doubt be true for live attenuated dengue vaccine virus strains as well. We chose to focus on EDIII as it would not only enable the elimination of prM and other parts of E implicated in ADE, it would also help focus the immune response to a functionally relevant epitope capable of eliciting potent DENV-nAbs. To compensate for the apparent non-immunodominance of EDIII, we displayed it on HBV S antigen-based VLP platform, and created mosaic VLPs displaying EDIIIs of all four DENV serotypes linked in tandem in a head-to-tail array, akin to the RTS,S vaccine for malaria [43, 44].

Though the AG129 mouse is susceptible to DENV infection and can manifest ADE, we opted to assess the immunogenicity of DSV4 in the BALB/c mouse. This is because the latter being immunocompetent is likely to reflect the immunogenicity of DSV4 more appropriately. Further, subunit vaccines tend to be less immunogenic in the AG129 mouse due to its genetic defect in the innate immune signaling pathway [59]. DSV4 formulated in alhydrogel adjuvant was immunogenic in BALB/c mice (as well as two other mouse strains), eliciting tetravalent seroconversion. Consistent with its design, DSV4 elicited antibodies to EDIIIs of all four DENV serotypes and to the HBV S antigen. Importantly, DSV4-elicited antibodies could neutralize the infectivity of all four DENVs. The nAb titers ranged from ~360 (DENV-4) to ~970 (DENV-3). These nAb titers are not insignificant given that it is conventionally accepted that titers >10 are protective. In this context, a longitudinal cohort study published recently found an inverse relationship between the probability of symptomatic DENV infection and serum nAb titers [60]. Further, the virus-neutralizing activity of the DSV4-induced antibodies spanned multiple genotypes. This breadth of virus neutralization across genotypes within each serotype is apparently the outcome of the polyclonal antibody response which is able to overcome the intra-serotypic variation among the genotypes. This also attests to the utility of EDIII as a vaccine immunogen whose serotype-specificity is conserved across genotypes. Though we tested only a total of 11 genotypes (representing the four DENV serotypes), it is noteworthy that the EDIII sequence of each DENV serotype represented in DSV4 shares ≥88% identity with the EDIII of the top 500 dengue sequences of the cognate serotype in the NCBI database (S3 Table).

Would nAbs elicited by DSV4 VLPs mediate ADE? It is known that even potent nAbs, if sufficiently diluted, can cause ADE *in vitro*. We could see evidence of homotypic enhancement in K562 cells using serially diluted anti-DSV4 antiserum. However, it is becoming increasingly apparent that *in vitro* ADE data obtained using FcγR-containing cells such as K-562 and THP-1, do not reflect *in vivo* ADE potential of serotype-specific nAbs. For example, the type-specific DENV-2 mAb 3H5 which at low doses can cause ADE in THP-1 cells did not enhance DENV-2 infection of AG129 mice [48]. Similarly, though polyclonal dengue-immune macaque serum enhanced infection of K562 cells by zika virus (ZIKV), a related flavivirus, ZIKV-challenged dengue-immune macaques did not experience infection enhancement [61]. Thus, rather than *in vitro* ADE data obtained using K562 cells, we hold that it is more appropriate to assess *in vivo* ADE potential using the AG129 model [57]. Nonetheless, the data need to be interpreted with caution as there have not been adequate studies to determine the correlation between *in vitro* ADE using a biologically relevant concentration of dengue immune sera (i.e., undiluted) with disease outcome in humans experiencing a post-primary dengue infection.

Remarkably, we found that anti-DSV4 antiserum dilutions high enough to result in virtually undetectable pre-challenge nAb titers did not cause ADE *in vivo*. This conclusion was further fortified by showing that sub-lethal doses of DENV-2 S221 neutralized *in vitro*, either partially or completely, with anti-DSV4 antiserum did not cause significant mortality when injected into AG129. This is consistent with a recent report which showed that partially neutralized DENV-2 ICs generated using EDIII-2-specific mAb 3H5 did not manifest significant ADE in AG129 mice in contrast to completely neutralized DENV-2 ICs generated using mAbs 4G2 and 6B6C-1, which target epitopes outside EDIII [48].

Interestingly, antisera against DENV-2 (whether in the passive transfer experiment in Fig 3D or in the IC inoculation experiment shown in Fig 4A) augmented a sub-lethal DENV-2 infection, resulting in ADE. This is intriguing because it represents ADE by homotypic antibodies. While this requires further study, it must be pointed out that DENV is potentially capable of eliciting large amounts of cross-reactive antibodies [15–17], presumably targeting epitopes outside EDIII as well as anti-prM antibodies, which can mediate ADE. Collectively, these data lead to the conclusion that anti-EDIII antibodies may lack ADE potential *in vivo* even at low concentrations. This represents an inherent safety attribute of the DSV4 vaccine candidate.

Though the AG129 mouse represents an immune-compromised model, its limitation in assessing DSV4 vaccine efficacy was somewhat overcome in that DSV4-induced antibodies were raised in immunocompetent BALB/c mice. Support for the notion that anti-DSV4 VLP-induced antibodies confer protection against potentially lethal DENV infection emanates from the following: (i) passive transfer of antibodies elicited by DSV4 VLPs significantly suppressed viremia upon sub-lethal challenge of AG129 mice with DENV-2 S221 (Fig 3B); (ii) AG129 mice challenged with a sub-lethal dose of DENV-2 S221 (‘NMS’ group) which show no signs of mortality for 2 weeks, succumb completely by 4 weeks (Fig 4A). This means that the sub-lethal dose over time has become lethal due to virus replication. High survival rates of AG129 mice inoculated with the same sub-lethal dose of DENV-2 S221 in the form of ICs (either partially or fully neutralized) generated *in vitro* with anti-DSV4 VLP antiserum well beyond 4 weeks, when all mice in the ‘NMS’ group have been killed, is noteworthy; (iii) anti-DSV4 antibodies also suppressed viremia in AG129 mice challenged with a lethal dose of DENV-4 strain 703–4 (Fig 5A). In addition, these anti-DSV4 antibodies conferred a significant level of protective efficacy against such lethal challenge (Fig 5B). DSV4 was also immunogenic in macaques, while requiring an additional adjuvant, eliciting nAbs to all four DENV serotypes, which did not manifest any *in vivo* ADE, using the AG129/sub-lethal DENV-2 S221 challenge model. We did not attempt to evaluate the protective efficacy or ADE potential of DSV4 in macaques at this stage due to ethical issues, biocontainment requirements and high cost.

Our data provide proof-of-concept supporting the *in vivo* efficacy of DSV4 and lack of ADE. These studies need to be extended to all serotypes. DSV4 is produced in yeast, which has a high expression potential. It elicits nAbs, with negligible ADE potential, against the functionally relevant serotype-specific EDIIIs. DSV4 may possibly help in circumventing potential ADE of ZIKV infection [62]. Indeed, recent work has shown that antibodies to DENV EDIII do not enhance ZIKV infection [63]. DSV4 is based on a VLP platform that is inherently highly immunogenic, with the S antigen providing T-cell help. In this context, it is relevant to note that prior HBV immunity in vaccine recipients may be beneficial, based on studies of the RTS,S malaria vaccine which found HBV seropositive recipients manifested earlier and efficient seroconversion to malarial antigen [64]. The unique four-in-one antigen design facilitates the expression and purification of a single tetravalent immunogen, as opposed to making four monovalent vaccines. These data support the promise of DSV4 as an alternate dengue vaccine candidate.

## Supporting information

S1 Appendix Supplementary materials and methods. (DOCX)

S1 Fig Design and expression of DSV4. (A) DS-pPICZA plasmid with *DS* gene cloned between the alcohol oxidase 1 (*AOX1*) promoter and the *AOX1* transcription terminator (T). The EDIII-1, EDIII-2, EDIII-3, EDIII-4 and S encoding regions of the *DS* gene which are in frame with each other are indicated by the magenta, green, blue, black and grey boxes, respectively. This 3.4 kb DS expression cassette (indicated by the white star) is flanked by *Bgl* II and *Bam* HI sites. (B) 4S-pAO815 plasmid carrying a 7.8 kb insert (indicated by the black star symbol) flanked by *Bgl* II and *Bam* HI sites. The 7.8 kb insert carries 4 copies of the S antigen expression cassette in a tandem head-to-tail array. (C) DSV4-pAO815 plasmid generated after insertion of 3.4 Kb *DS* gene expression cassette into the *Bam* HI site of the 4S-pAO815 plasmid. The DSV4 part (indicated by the white and black star symbols) of this construct is represented by the 11.2 Kb sequence flanked by *Bgl* II and *Bam* HI sites. Maps are not drawn to scale. (D) Agarose gel analysis of the three plasmids in ‘A’ (lane 2), ‘B’ (lane 3) and ‘C’ (lane 4), following double digestion with *Bgl* II and *Bam* HI. Fragment lengths of *DS* (3.4 kb), *S* (7.8 kb) and *DSV4* (11.2 kb) expression cassettes are indicated by white, black, and combination of white and black stars, respectively. (E) Whole cell lysates (lanes 1 & 4), supernatants (lanes 2 & 5) and solubilized pellets (lanes 3 & 6) of un-induced (lanes 1–3) and induced (lanes 4–6) cells obtained after lysis in native buffer were analyzed for the presence of DSV4 by silver staining. Low molecular weight protein markers were analyzed in lane ‘M’. Their sizes (kDa) are shown on the left. Positions of DS, S dimer and S are indicated on the right. (F) Same samples as in ‘E’ analyzed in a Western blot with HBsAg specific antibody. Pre-stained protein size markers were analyzed in lane ‘M’. Their sizes (kDa) are shown on the left. Positions of DS, S dimer and S are indicated on the right. (G) Time-dependent expression-optimization of DSV4 induced at fixed methanol concentration (2%). Aliquots of 1ml induced culture were collected at indicated times and the amount of DSV4 was quantified by ELISA with HBsAg specific antibody. (H) Methanol-dependent expression-optimization of DSV4 induced for fixed duration (72 hours). DSV4 was quantified as in ‘G’, following induction using methanol at the indicated concentrations.
(TIF)

S2 Fig Purification and initial characterization of DSV4. (A) Purification profile of DSV4 using hydrophobic interaction chromatography on phenyl-600M. Blue and black lines have their corresponding *y*-axis in blue and black color, respectively. The solid blue line represents the elution profile monitored by absorbance at 280 nm. Black dashed and dotted lines represent pH and urea concentration, respectively. Quality of protein eluted in each of the four peaks (1, 2, 3 and 4) was analyzed by silver stained SDS-PAGE (inset). (B) DSV4 fractions from peaks 3 and 4 (panel A) were pooled and analyzed by silver stain (lane 1), Western blots with HBV S specific antibody (lane 2) and in-house dengue specific antibody (lane 3). (C) Three batches (I, II and III) of purified and dialyzed DSV4 at three different concentrations (lane 1: 1.2 μg; lane 2: 0.6 μg and lane 3: 0.3 μg) were analyzed in Western blots using anti-HBV S antigen-specific mAb. Positions of DS, S dimer and S (in panel A inset, panels B and C) are indicated on the right by the upper, middle and lower arrows, respectively. Protein markers were run in lanes ‘M’; their sizes (in kDa) are indicated on the left of inset in panel A and panel B. Pre-stained protein markers were run in lane M of panel C; their sizes (in kDa) are indicated on the left of the batch I panel. Band intensities in the blots were quantified by densitometric scanning. Ratio of DS/S was calculated for each of the three loads of all the batches to determine the average DS/S ratio of each DSV4 batch (Table adjacent to panel ‘C’).
(TIF)

S3 Fig Evaluation of consistency in DSV4 production and its stability to storage under different conditions. (A) Three separate batches of DSV4 (I, II, III) were analyzed for consistency in yield, VLP size (by DLS) and purity (by densitometry). (B) Silver-stained SDS-PAGE of the three batches of DSV4. Protein markers were run in lanes 'M' and their sizes (in kDa) are indicated on the left of the first gel image (with the marker positions indicated by corresponding dots to the left of the second and third gel images). The positions of DS, S and S dimer are indicated by the upper, lower and middle arrows, respectively, to the right of the third gel image. (C) Sandwich ELISA reactivity of the three batches of DSV4 with DENV-1 (magenta), DENV-2 (green), DENV-3 (blue) and DENV-4 (black) -specific EDIII mAbs. The data shown are average (*n* = 2) ELISA absorbance (O.D.) values. (D) Schematic representation of the sandwich ELISA format used with monoclonal antibody specific to EDIII (EDIII mAb) to capture and mAb specific to S (anti-HBV S-HRPO) to reveal the captured antigen. (E) Silver stained gels of three DSV4 batches (I, II and III) stored at 4°C for 1, 2, 3 and 6 months. Each batch was run in triplicates. The positions of DS, S and S dimer are indicated by the upper, lower and middle arrows, respectively, on the right side of the ‘6^th^ month’ panel. Molecular weight markers were run in lanes 'M'; their sizes (in kDa) are indicated on the left of each panel. (F) Silver stained gel of batch I stored at 25°C (lane 1), -20°C (lane 2), -80°C (lane 3) and in liquid nitrogen (lane 4) for a month. Further aliquots of DSV4 were stored at 25°C for 1h (lanes 5, 10), 2h (lanes 6, 11), 4h (lanes 7, 11), 8h (lanes 8, 13) and 24h (lanes 9, 14). Samples were centrifuged and supernatant (lane 5–9) and pellet (lane 10–14) fractions were separated and analyzed. Storage of DSV4 at 25°C was further extended with aliquots collected on days 3 (lane 15) and 5 (lane 16), and the pellets after centrifugation were analyzed on silver-stained SDS-PAGE. The positions of DS, S and S dimer are indicated by the upper, lower and middle arrows, respectively, on the right side. Molecular weight markers were run in lane 'M'; their sizes (in kDa) are indicated on the left.
(TIF)

S4 Fig DSV4-induced DENV-specific antibody titers in macaques. (A) Groups of six macaques (macaque numbers 1–6 on *x*-axis) each were immunized either with DSV4/alhydrogel (top panel) or DSV4/alhydrogel + MPLA (bottom panel) on days 0, 30 and 84. Individual sera collected two weeks after the final immunization (day 98) were analyzed by indirect ELISA using the following 5 recombinant proteins as coating antigens: EDIII-1 (magenta); EDIII-2 (green); EDIII-3 (blue), EDIII-4 (black) and S (grey) proteins. Data shown are the averages of two separate experiments. (B) Sera from macaques, immunized as described in ‘A’ (top panel: DSV4/alhydrogel; bottom panel: DSV4/alhydrogel + MPLA) were collected on days 42, 84, 98 and 119 (*x*-axis) and FNT_50_ titers (*y*-axis) against the four DENV serotypes (DENV-1: magenta; DENV-2: green; DENV-3: blue; and DENV-4: black) were determined separately for each of the six macaques (represented by the six panels in each row). Each data point represents the average of two separate experiments.
(TIF)

S1 Table Purification of DSV4 from 50 g induced biomass. (DOCX)

S2 Table Stability of DSV4 stored under different conditions. (DOCX)

S3 Table Comparison of multiple EDIIIs within each serotype with the EDIII sequences used to design DSV4. (DOCX)

S4 Table FNT_50_ titers in macaque anti-DSV4 antiserum before and after depletion of EDIII-specific antibodies. (DOCX)
