# Supplementary material for: A tetravalent virus-like particle vaccine designed to display domain III of dengue envelope proteins induces multi-serotype neutralizing antibodies in mice and macaques which confer protection against antibody dependent enhancement in AG129 mice
Source: PLoS Negl Trop Dis. 2018 Jan 8;12(1):e0006191. doi: 10.1371/journal.pntd.0006191 (PMC5774828; doi:10.1371/journal.pntd.0006191)
Supplement: S1 Appendix — (DOCX) [file pntd.0006191.s001.docx]

**S1 Appendix**

**Supplementary materials**

**Cell lines, DENVs, anti-EDIII serotype-specific mAbs and other reagents**

Vero cells were from American Type Culture Collection (ATCC), Virginia, USA. *E. coli* clones expressing MBP, EDIII-1-MBP, EDIII-2-MBP and EDIII-3-MBP were kindly provided by Dr. Aravinda de Silva, University of North Carolina, USA. The WHO reference strains of DENV-1 (West-Pac 74), DENV-2 (S-16803), DENV-3 (CH53489) and DENV-4 (TVP-360) have been reported earlier (*47*). Different strains of DENV-1 (UNC 1036, West Pac 74 and UNC 1017), DENV-2 (UNC2037), DENV-3 (UNC 3043 and UNC 3001) and DENV-4 (UNC 4019) were kindly provided by Dr. Aravinda de Silva. DENV-2 strains S-16803 and IQT2133, DENV-3 strain CH53489 and DENV-4 strain TVP 360 were provided by Dr. Jorge Muñoz, Center for Diseases Control, Atlanta, USA. DENV-2 S221 and DENV-4 703-4 were kindly provided by Dr. Sujan Shresta, La Jolla Institute for Allergy and Immunology, CA, USA, and Dr. Alan Barrett, University of Texas Medical Branch, Galveston, TX, USA), respectively.

All EDIII-specific mAbs used in this study have been reported earlier (*16, 21, 29, 49-55*). Adjuvants CpG DNA and MPLA were procured from InvivoGen, San Diego, California, USA, while alhydrogel was obtained from Brenntag Biosector, Denmark. The HBV S antigen was expressed using *P. pastoris* and purified in-house as described previously (*40*).

**Supplementary methods**

**Recombinant DSV4 expression and localization**

Yeast cultures were grown at 30°C in buffered glycerol-containing medium (BMGY). At log phase of growth, BMGY was replaced with methanol-containing buffered medium to induce DSV4 expression. In preliminary expression studies, a range of methanol concentrations for fixed time duration, and at fixed methanol concentration for various induction durations were evaluated. Based on this, routine inductions were performed at 2% methanol (vol/vol) for 72 hours. Total (T) lysates were prepared from induced cells using glass beads in a detergent-containing buffer as done earlier (*40*). For localization studies, a portion of the T lysate was centrifuged to obtain the supernatant ‘S’ fraction and the membrane-enriched pellet ‘P’ fraction. DSV4 in the ‘T’, ‘S’ and urea-solubilized ‘P’ fractions were analyzed by SDS-PAGE followed by silver staining, Western blotting and ELISA using EDIII-specific mAb24A12 and S antigen-specific mAb 5S (*40*).

**Purification of recombinant DSV4**

Induced biomass (~50 g) obtained after 3 days of 2% methanol-induction was re-suspended in 400 ml ice-cold suspension buffer (20 mM sodium phosphate, pH 7.2/150 mM NaCl/5 mM EDTA/8% glycerol) and lysed in Dyno-mill (WAB, Muttenz, Switzerland) using a flow rate of 100 ml/min. The resultant lysate was centrifuged at 16,000 x*g* to separate the membrane-rich ‘P’ fraction, which was extracted in 200 ml of extraction buffer (2% Tween 20/8 M Urea/20 mM sodium phosphate, pH 7.2/150 mM NaCl) for 2 hours at room temperature (RT). This extract was diluted 4-fold with PBS (20 mM sodium phosphate, pH 7.2/150 mM NaCl) and stirred overnight with 5% polyethylene glycol (PEG)-6000 at 4ºC. This material was centrifuged at 16,000 x*g* and supernatant obtained was subjected to tangential flow filtration (TFF) across 300 kDa cut-off membrane (Millipore, Billerica, USA), using sequentially 4 L each of 2 M, 1 M and 0 M urea in PBS, to allow gradual removal of urea and the detergent. Next, the TFF retentate was loaded onto a Phenyl-600M Toyopearl resin (TOSOH, Tokyo, Japan) column (100 ml bed volume) at RT. The column was washed with 4 column volumes (CV) of 20 mM sodium phosphate, pH7.2, followed by 4 CV 20 mM sodium bicarbonate buffer, pH 9.6 (BB). Elution was performed in two steps with 4 CV BB containing 2 M urea and 4 CV BB containing 6 M urea. Eluted protein fractions were analyzed by SDS-PAGE followed by silver staining and pure fractions were pooled and dialyzed against PBS, pH 7.2, at 4^o^C. Total yield and specific activity of DSV4 at each step of purification was determined using Bicinchoninic Acid (BCA) assay (Pierce, Thermo Fischer Scientific, USA) and Hepanostika® HBsAg Ultra micro ELISA (BioMérieux, Marcy-l'Étoile, France), respectively.

**Sandwich ELISA**

Antigenic integrity of EDIII epitopes on DSV4 VLPs was assessed using a sandwich ELISA (Fig S4D). In this assay, DSV4 VLPs were captured using a panel of different serotype-, sub-complex- and complex-specific anti-DENV mAbs and revealed using anti-S antigen mAb horse radish peroxidase (HRPO) conjugate (from Hepnostika® HBsAg Ultra micro ELISA kit). Briefly, anti-EDIII mAbs were coated (50 ng/100 µl/well in 100 mM sodium bicarbonate, pH 9.6) on polystyrene ELISA plates (Costar, Corning Incorporated, NY, USA) overnight at 4ºC. Wells were washed 3 times with wash buffer (137 mM NaCl/2.7 mM KCl/10 mM Na_2_HPO_4_/1.8 mM KH_2_PO_4_/0.1% Tween-20, pH 7.2) and blocked with 5% skim milk in 1x phosphate buffered saline, PBS (137 mM NaCl/2.7 mM KCl/10mM Na_2_HPO_4_/1.8mM KH_2_PO_4_) for 2 hours at 37 ºC. After blocking, wells were washed again as before (3x with wash buffer) and incubated overnight with DSV4 (1 µg/100 µl/well) in protein dilution buffer (20 mM sodium phosphate, pH 7.2/150 mM NaCl). Next, wells were washed (6 times) and incubated with reveal antibody conjugate (Hepnostika kit anti-S-HRPO conjugate, specimen diluent and protein dilution buffer mixed in 2:1:1 ratio) for 1 hour at 37°C. This was followed by washing the wells (6 times) and the addition of 100 µl/well HRPO substrate, 3, 3′, 5, 5′-Tetramethylbenzidine (Sigma-Aldrich, St. Louis, MO, USA). After 30 minutes of RT incubation with the substrate, the reaction was stopped using 1 M H_2_SO_4_ (100 µl/well). Plates were read for absorbance at 450 nm. To score the reactivity of each anti-DENV mAb towards DSV4 as positive or negative in the sandwich ELISA, a baseline was established by taking the mean ELISA absorbance of each of these mAbs towards S protein as an irrelevant antigen and adding three times the SD to it to obtain a cut-off absorbance value of 0.275. ELISA absorbance of a given mAb above or below this cut-off was taken as indication of the presence or absence, respectively, of the cognate epitope on DSV4.

**Indirect ELISA**

Indirect ELISA was performed using five purified recombinant proteins, EDIII-1, EDIII-2, EDIII-3, EDIII-4 and S antigens, to capture cognate antibodies from anti-DSV4 antiserum and revealed using anti-mouse IgG-HRPO conjugate. To begin with, 500 ng of purified antigen was coated per well in coating buffer (0.1 M sodium bicarbonate buffer, pH 9.6) overnight at 4°C. Next, wells were washed three times with 1x PBS containing 1% Tween-20 (1x PBS-T) wash buffer and blocked with 5% skim milk made in 1x PBS at 37°C for 2 hours. Wells were washed three times with wash buffer. Next, serial two-fold dilutions of murine immune sera were added to the wells (100 μl/well) and incubated at 37°C for 1 hour. This was followed by washing the wells six times with wash buffer and incubation with 100 μl/well of anti-mouse IgG (H&L chain specific) HRPO conjugate (0.1μg/ml in 1x PBS with 2.5% skim milk) at 37°C for 1 hour. Wells were washed six times and incubated with 100 μl/well of TMB (soluble) substrate at 37°C for 30 minutes. The reaction was stopped by adding 1N H_2_SO_4_ (100 μl/well) and the absorbance read at 450 nm.

**Antibody depletion of anti-DSV4 antisera**

A portion of the pooled immune serum from DSV4-immunized BALB/c mice was subjected to pre-depletion of EDIII-specific antibodies on immobilized maltose-binding protein (MBP)-EDIII-2 and MBP-EDIII-3 as follows. In-house purified MBP and MBP-EDIII fusion proteins (0.3 mg each) were bound separately to 100 µl aliquots of amylose resin (in 20 mM Tris-HCl/200 mM NaCl/1 mM EDTA, pH 7.4) by incubating overnight at 4^o^C. The resin containing bound protein was washed with 1x PBS, blocked with 1% bovine serum albumin in 1x PBS (2 hours at 37^o^C), washed with 1x PBS again and incubated with 1 ml of 10-fold diluted immune serum (45 minutes at 37^o^C). Depleted antiserum was recovered by centrifugation of the suspension at 3,000 rpm (5 minutes at RT) and used for the determination anti-DENV nAb titers using the FACS assay. A serum aliquot mock-depleted on immobilized MBP served as the reference.

***In vivo* ADE assay & cytokine analysis**

Murine or macaque antisera (200-250 μl) were passively transferred into 6-8 week old AG129 mice *via* intraperitoneal (i.p.) injection 24 hours in advance (day -1). The next day (day 0), these mice were challenged intravenously (i.v.) with a sub-lethal dose of DENV-2 S221 (5x10^3^ FIU). A small amount of blood was collected ~2 hours before challenge to measure pre-challenge FNT_50_ titers. On day 3, sera were collected for viral RNA quantification by reverse transcription qPCR. In some experiments, rather than prior passive transfer, AG129 mice were directly challenged with *in vitro* neutralized DENV-2 S221/antibody immune complex (IC) as described below. The IC complexes were generated by mixing 2x10^4^ FIU of DENV-2 S221 with appropriately diluted anti-DSV4 antiserum (equivalent to 30% or 100% neutralization) in a total volume of 50 μl and incubating for 1 hour on ice. This was then injected i.v (retro-orbital) into 6-8 week old AG129 mouse (*n*=9). A sub-set (*n*=3) of IC-inoculated mice were euthanized on day 3 post-IC inoculation and perfused extensively with 1x PBS to remove all visible luminal content. The small intestines were collected and quick frozen in liquid nitrogen until extraction. To prepare extract for cytokine estimation, the tissue was homogenized in 1x PBS using a Polytron homogenizer and centrifuges. The clear supernatant was used for the ELISA-based determination of TNF-α (cat# KMC3011) and IL-6 (cat# KMC0061) using commercial kits purchased from Invitrogen, against recombinant biotinylated murine TNF-α and IL-6, respectively, as reference standards. In the remaining animals, mortality/morbidity was monitored for up to 45 days, and were scored 0.5-4 on the basis of symptoms as follows: 0.5, mild ruffled fur; 1, ruffled fur; 1.5, loose stools, eyes compromised; 2, lethargy; 2.5, limited mobility from stimulation, hunching; 3, not moving, or >20% initial weight loss; 4, moribund. Mice were euthanized when clinical score was 3 or higher.
