# Supplementary figures and images for: A tetravalent virus-like particle vaccine designed to display domain III of dengue envelope proteins induces multi-serotype neutralizing antibodies in mice and macaques which confer protection against antibody dependent enhancement in AG129 mice
Source: PLoS Negl Trop Dis. 2018 Jan 8;12(1):e0006191. doi: 10.1371/journal.pntd.0006191 (PMC5774828; doi:10.1371/journal.pntd.0006191)

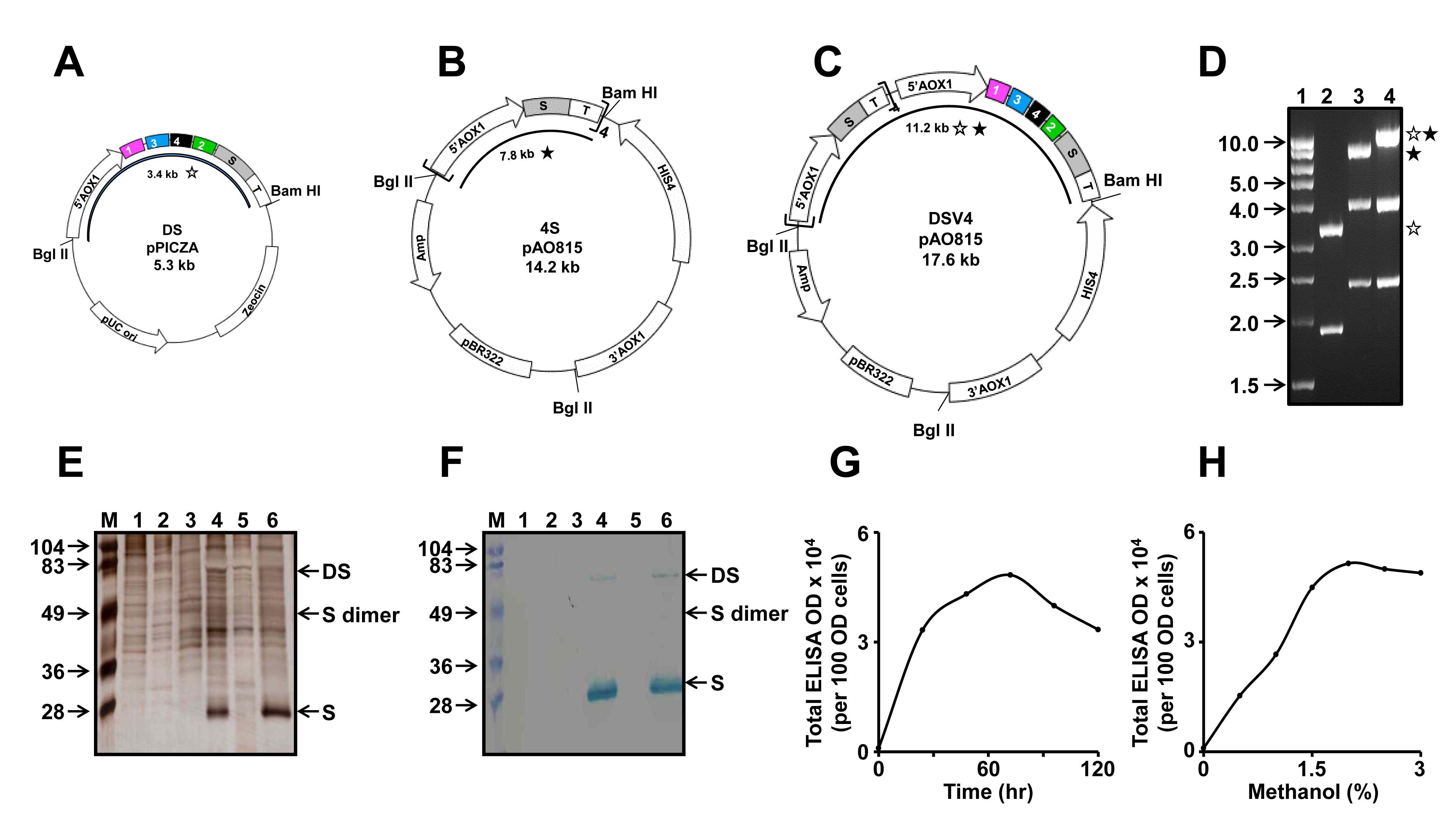

Supplement: S1 Fig — (A) DS-pPICZA plasmid with DS gene cloned between the alcohol oxidase 1 (AOX1) promoter and the AOX1 transcription terminator (T). The EDIII-1, EDIII-2, EDIII-3, EDIII-4 and S encoding regions of the DS gene which are in frame with each other are indicated by the magenta, green, blue, black and grey boxes, respectively. This 3.4 kb DS expression cassette (indicated by the white star) is flanked by Bgl II and Bam HI sites. (B) 4S-pAO815 plasmid carrying a 7.8 kb insert (indicated by the black star symbol) flanked by Bgl II and Bam HI sites. The 7.8 kb insert carries 4 copies of the S antigen expression cassette in a tandem head-to-tail array. (C) DSV4-pAO815 plasmid generated after insertion of 3.4 Kb DS gene expression cassette into the Bam HI site of the 4S-pAO815 plasmid. The DSV4 part (indicated by the white and black star symbols) of this construct is represented by the 11.2 Kb sequence flanked by Bgl II and Bam HI sites. Maps are not drawn to scale. (D) Agarose gel analysis of the three plasmids in ‘A’ (lane 2), ‘B’ (lane 3) and ‘C’ (lane 4), following double digestion with Bgl II and Bam HI. Fragment lengths of DS (3.4 kb), S (7.8 kb) and DSV4 (11.2 kb) expression cassettes are indicated by white, black, and combination of white and black stars, respectively. (E) Whole cell lysates (lanes 1 & 4), supernatants (lanes 2 & 5) and solubilized pellets (lanes 3 & 6) of un-induced (lanes 1–3) and induced (lanes 4–6) cells obtained after lysis in native buffer were analyzed for the presence of DSV4 by silver staining. Low molecular weight protein markers were analyzed in lane ‘M’. Their sizes (kDa) are shown on the left. Positions of DS, S dimer and S are indicated on the right. (F) Same samples as in ‘E’ analyzed in a Western blot with HBsAg specific antibody. Pre-stained protein size markers were analyzed in lane ‘M’. Their sizes (kDa) are shown on the left. Positions of DS, S dimer and S are indicated on the right. (G) Time-dependent expression-optimization of DSV4 [file pntd.0006191.s002.tif]

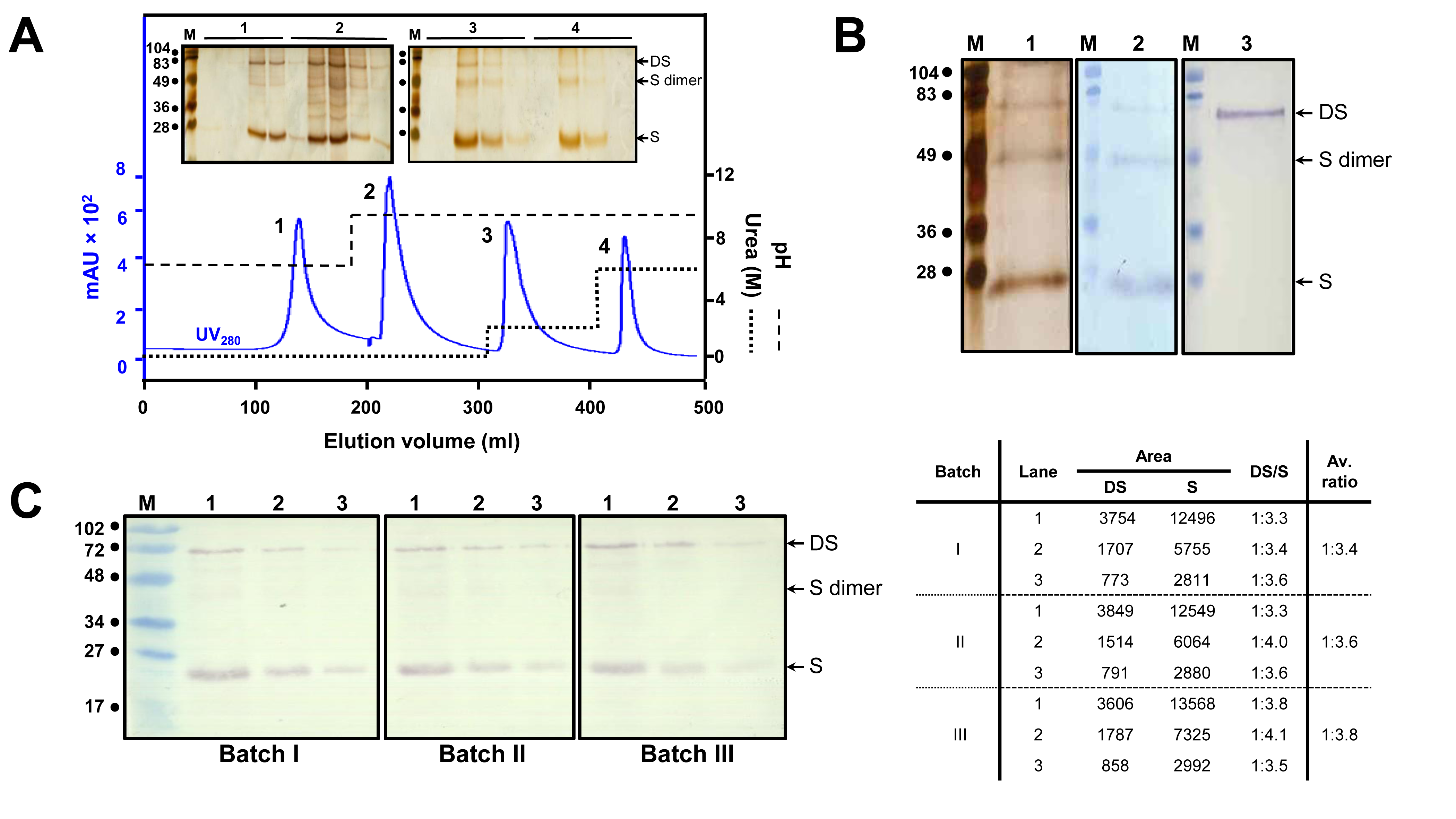

Supplement: S2 Fig — (A) Purification profile of DSV4 using hydrophobic interaction chromatography on phenyl-600M. Blue and black lines have their corresponding y-axis in blue and black color, respectively. The solid blue line represents the elution profile monitored by absorbance at 280 nm. Black dashed and dotted lines represent pH and urea concentration, respectively. Quality of protein eluted in each of the four peaks (1, 2, 3 and 4) was analyzed by silver stained SDS-PAGE (inset). (B) DSV4 fractions from peaks 3 and 4 (panel A) were pooled and analyzed by silver stain (lane 1), Western blots with HBV S specific antibody (lane 2) and in-house dengue specific antibody (lane 3). (C) Three batches (I, II and III) of purified and dialyzed DSV4 at three different concentrations (lane 1: 1.2 μg; lane 2: 0.6 μg and lane 3: 0.3 μg) were analyzed in Western blots using anti-HBV S antigen-specific mAb. Positions of DS, S dimer and S (in panel A inset, panels B and C) are indicated on the right by the upper, middle and lower arrows, respectively. Protein markers were run in lanes ‘M’; their sizes (in kDa) are indicated on the left of inset in panel A and panel B. Pre-stained protein markers were run in lane M of panel C; their sizes (in kDa) are indicated on the left of the batch I panel. Band intensities in the blots were quantified by densitometric scanning. Ratio of DS/S was calculated for each of the three loads of all the batches to determine the average DS/S ratio of each DSV4 batch (Table adjacent to panel ‘C’). (TIF) [file pntd.0006191.s003.tif]

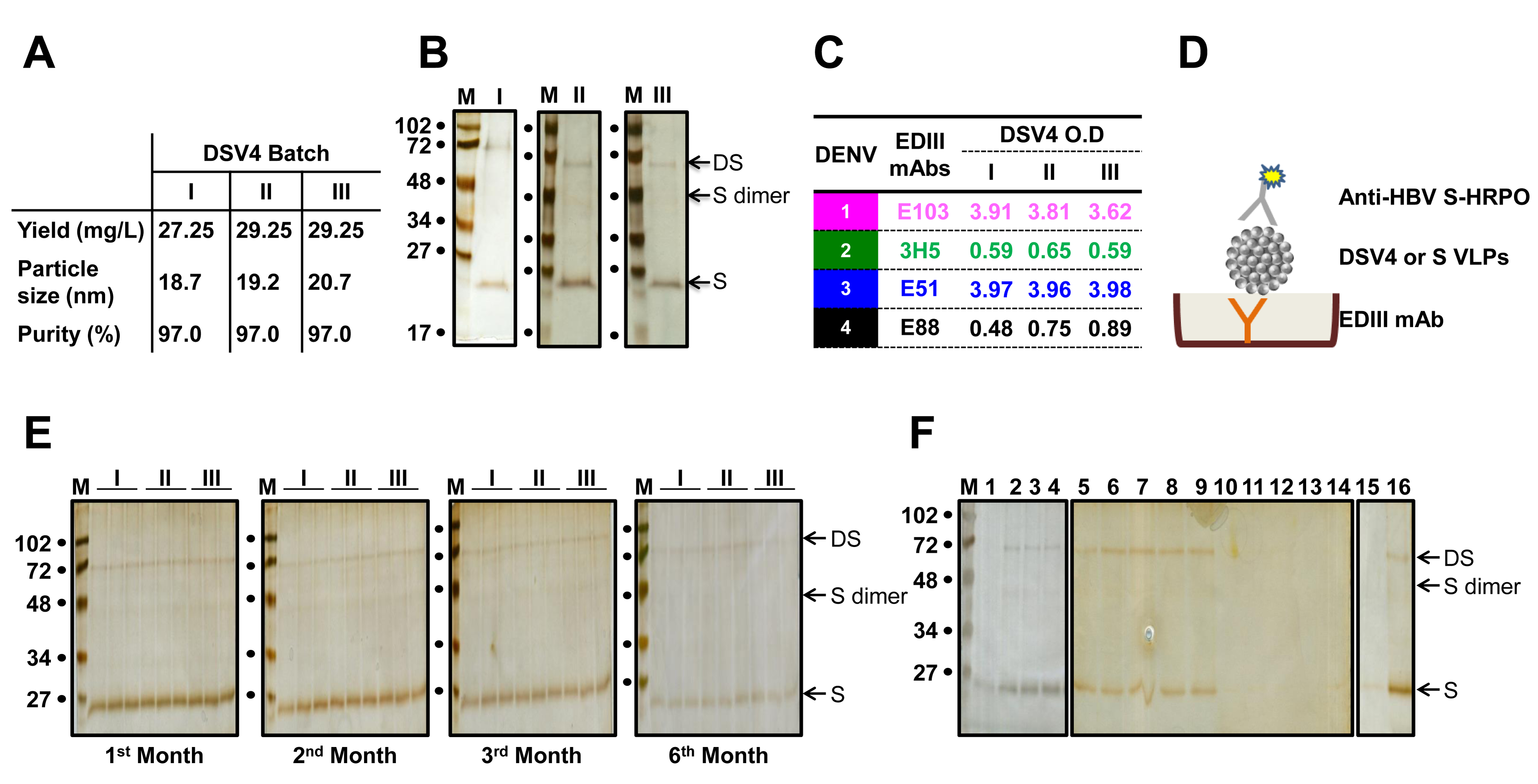

Supplement: S3 Fig — (A) Three separate batches of DSV4 (I, II, III) were analyzed for consistency in yield, VLP size (by DLS) and purity (by densitometry). (B) Silver-stained SDS-PAGE of the three batches of DSV4. Protein markers were run in lanes 'M' and their sizes (in kDa) are indicated on the left of the first gel image (with the marker positions indicated by corresponding dots to the left of the second and third gel images). The positions of DS, S and S dimer are indicated by the upper, lower and middle arrows, respectively, to the right of the third gel image. (C) Sandwich ELISA reactivity of the three batches of DSV4 with DENV-1 (magenta), DENV-2 (green), DENV-3 (blue) and DENV-4 (black) -specific EDIII mAbs. The data shown are average (n = 2) ELISA absorbance (O.D.) values. (D) Schematic representation of the sandwich ELISA format used with monoclonal antibody specific to EDIII (EDIII mAb) to capture and mAb specific to S (anti-HBV S-HRPO) to reveal the captured antigen. (E) Silver stained gels of three DSV4 batches (I, II and III) stored at 4°C for 1, 2, 3 and 6 months. Each batch was run in triplicates. The positions of DS, S and S dimer are indicated by the upper, lower and middle arrows, respectively, on the right side of the ‘6th month’ panel. Molecular weight markers were run in lanes 'M'; their sizes (in kDa) are indicated on the left of each panel. (F) Silver stained gel of batch I stored at 25°C (lane 1), -20°C (lane 2), -80°C (lane 3) and in liquid nitrogen (lane 4) for a month. Further aliquots of DSV4 were stored at 25°C for 1h (lanes 5, 10), 2h (lanes 6, 11), 4h (lanes 7, 11), 8h (lanes 8, 13) and 24h (lanes 9, 14). Samples were centrifuged and supernatant (lane 5–9) and pellet (lane 10–14) fractions were separated and analyzed. Storage of DSV4 at 25°C was further extended with aliquots collected on days 3 (lane 15) and 5 (lane 16), and the pellets after centrifugation were analyzed on silver-stained SDS-PAGE. The positions of DS, S and S dimer are indicated by the [file pntd.0006191.s004.tif]

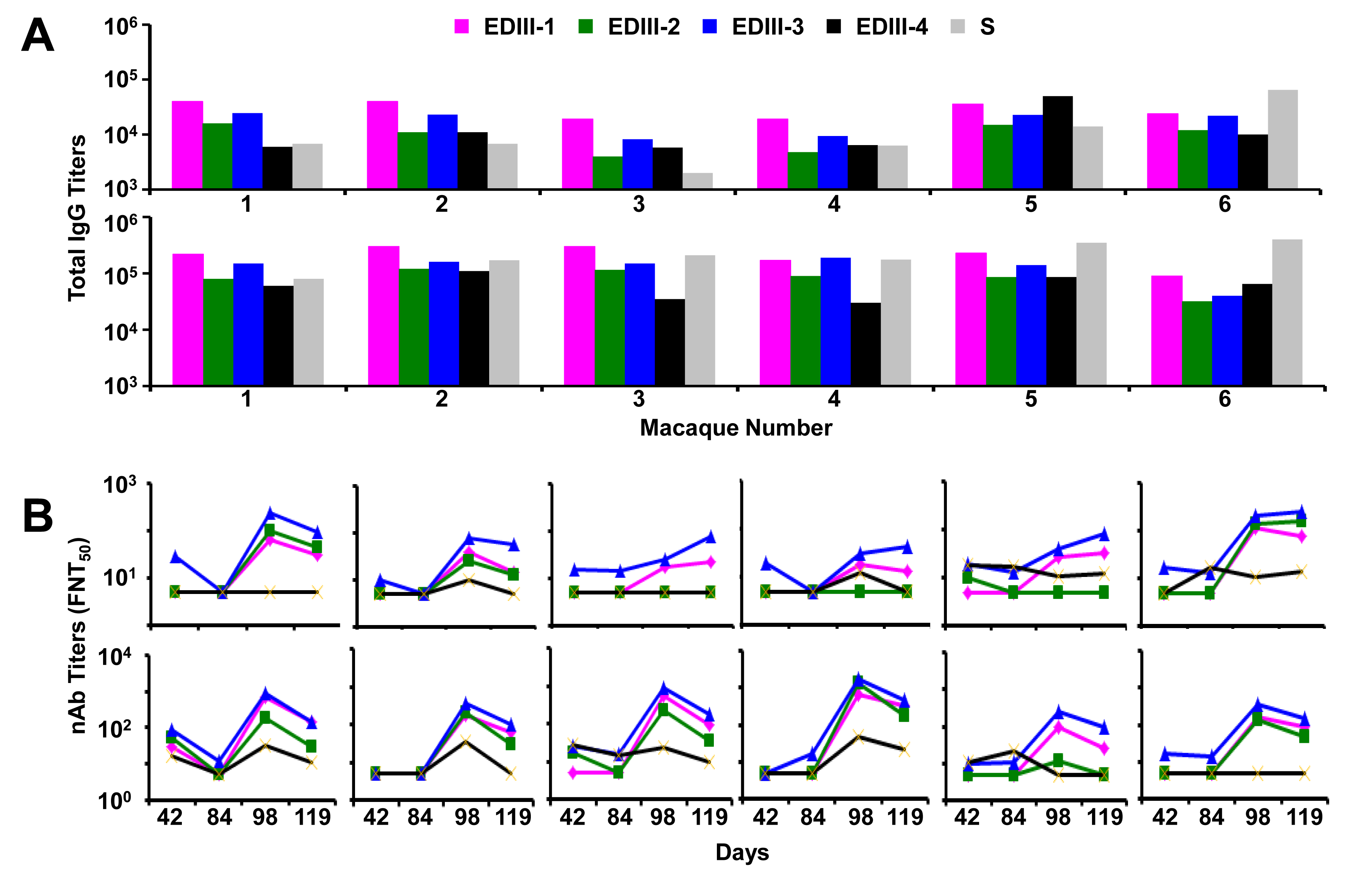

Supplement: S4 Fig — (A) Groups of six macaques (macaque numbers 1–6 on x-axis) each were immunized either with DSV4/alhydrogel (top panel) or DSV4/alhydrogel + MPLA (bottom panel) on days 0, 30 and 84. Individual sera collected two weeks after the final immunization (day 98) were analyzed by indirect ELISA using the following 5 recombinant proteins as coating antigens: EDIII-1 (magenta); EDIII-2 (green); EDIII-3 (blue), EDIII-4 (black) and S (grey) proteins. Data shown are the averages of two separate experiments. (B) Sera from macaques, immunized as described in ‘A’ (top panel: DSV4/alhydrogel; bottom panel: DSV4/alhydrogel + MPLA) were collected on days 42, 84, 98 and 119 (x-axis) and FNT50 titers (y-axis) against the four DENV serotypes (DENV-1: magenta; DENV-2: green; DENV-3: blue; and DENV-4: black) were determined separately for each of the six macaques (represented by the six panels in each row). Each data point represents the average of two separate experiments. (TIF) [file pntd.0006191.s005.tif]
