# Supplementary material for: A tetravalent virus-like particle vaccine designed to display domain III of dengue envelope proteins induces multi-serotype neutralizing antibodies in mice and macaques which confer protection against antibody dependent enhancement in AG129 mice
Source: PLoS Negl Trop Dis. 2018 Jan 8;12(1):e0006191. doi: 10.1371/journal.pntd.0006191 (PMC5774828; doi:10.1371/journal.pntd.0006191)
Supplement: S1 Table — (DOCX) [file pntd.0006191.s006.docx]

**S1 Table: Purification of DSV4 from 50 g induced biomass**^a^

| **Purification step** | **Total protein*^b^* (mg)** | **Total ELISA*^c^* O.D. (x10^5^)** | **Specific activity*^d^* (x10^2^)** | **Recovery*^e^* (%)** |
| --- | --- | --- | --- | --- |
| Cell lysate | 2181 | 172 | 79 | 100 |
| Membrane extract | 1309 | 168 | 128 | 98 |
| Supernatant (post poly ethylene glycol precipitation) | 850 | 159 | 187 | 92 |
| Diafiltration retentate  (300 kDa) | 400 | 120 | 300 | 70 |
| Phenyl 600M (pooled eluate) | 45 | 25 | 556 | 15 |

*^a^*Typical data from one of three purifications carried out

*^b^*Determined using BCA assay

*^c^*Determined using HBV S antigen-specific ELISA

*^d^*Ratio of total ELISA OD to total protein

*^e^*Determined with respect to cell lysate based on total ELISA OD
