# Supplementary material for: A tetravalent virus-like particle vaccine designed to display domain III of dengue envelope proteins induces multi-serotype neutralizing antibodies in mice and macaques which confer protection against antibody dependent enhancement in AG129 mice
Source: PLoS Negl Trop Dis. 2018 Jan 8;12(1):e0006191. doi: 10.1371/journal.pntd.0006191 (PMC5774828; doi:10.1371/journal.pntd.0006191)
Supplement: S2 Table — (DOCX) [file pntd.0006191.s007.docx]

**S2 Table: Stability of DSV4 Stored Under Different Conditions***^a^*

| **DENV**  **Serotype** | **EDIII mAb***^b^* | **1 mo** | | | | | **2 mo** | **3 mo** | **6 mo** |
| --- | --- | --- | --- | --- | --- | --- | --- | --- | --- |
|  |  | **Liq N_2_** | **-80^o^C** | **-20^o^C** | **4^o^C** | **25^o^C** | **4^o^C** | **4^o^C** | **4^o^C** |
| 1 | E103 | 3.40 | 3.43 | 3.40 | 3.47 | 0.33 | 3.07 | 2.47 | 0.20 |
| 2 | 3H5 | 0.43 | 0.50 | 0.40 | 0.50 | 0.13 | 0.43 | 0.27 | 0.10 |
| 3 | E51 | 3.37 | 3.23 | 3.07 | 3.47 | 0.83 | 3.13 | 2.50 | 0.20 |
| 4 | E88 | 0.50 | 0.53 | 0.50 | 0.50 | 0.10 | 0.47 | 0.33 | 0.10 |

*^a^*Aliquots of DSV4 (stored under different conditions as indicated on the top) were centrifuged and supernatants were analyzed by the sandwich ELISA using anti-EDIII mAbs specific to DENV-1 (magenta), DENV-2 (green), DENV-3 (blue) and DENV-4 (black). Values shown are ELISA absorbance values (mean of two determinations).

*^b^*These serotype-specific anti-EDIII mAbs are described in the following references: E103, Shrestha *et al*, 2010 [29]; 3H5, Henchal *et al*, 1982 [49]; E51, Brien *et al*, 2010 [51]; E88, Sukupolvi-Petty *et al*, 2013 [55].
