# Supplementary material for: A tetravalent virus-like particle vaccine designed to display domain III of dengue envelope proteins induces multi-serotype neutralizing antibodies in mice and macaques which confer protection against antibody dependent enhancement in AG129 mice
Source: PLoS Negl Trop Dis. 2018 Jan 8;12(1):e0006191. doi: 10.1371/journal.pntd.0006191 (PMC5774828; doi:10.1371/journal.pntd.0006191)
Supplement: S3 Table — (DOCX) [file pntd.0006191.s008.docx]

**S3 Table: Comparison of multiple EDIIIs within each serotype with the EDIII sequences used to design DSV4***^a^*

| **DSV4 EDIIIs** | **Specific DENV strain** | **Identity (%)** |
| --- | --- | --- |
|  |  |  |
| EDIII-1 | Nauru/West Pac/1974 | 95-100 |
| EDIII-2 | Puerto Rico/PR159-S1/1969 | 94-100 |
| EDIII-3 | Philippines/H87/1956 | 94-100 |
| EDIII-4 | H241-P | 88-100 |

*^a^*DENV EDIII sequences used in designing DSV4 were aligned with the top 500 available dengue sequences for each serotype in the NCBI data base (BLASTP 2.2.28+) to obtain the range of percent identity with members of the given serotype.
