# Supplementary material for: A tetravalent virus-like particle vaccine designed to display domain III of dengue envelope proteins induces multi-serotype neutralizing antibodies in mice and macaques which confer protection against antibody dependent enhancement in AG129 mice
Source: PLoS Negl Trop Dis. 2018 Jan 8;12(1):e0006191. doi: 10.1371/journal.pntd.0006191 (PMC5774828; doi:10.1371/journal.pntd.0006191)
Supplement: S4 Table — (DOCX) [file pntd.0006191.s009.docx]

**S4 Table: FNT_50_ titers in macaque anti-DSV4 antiserum before and after depletion of EDIII-specific antibodies**

| **Serum*^a^*** | **DENV-1** | **DENV-2** | **DENV-3** | **DENV-4** |
| --- | --- | --- | --- | --- |
| Un-depleted | 1446*^b^* | 209 | 710 | 118 |
| MBP | 1308 | 187 | 448 | 89 |
| MBP-EDIII-1 | 445 | 77 | 170 | 84 |
| MBP-EDIII-2 | 991 | 74 | 335 | 80 |
| MBP-EDIII-3 | 1082 | 99 | 63 | 87 |

*^a^*Pooled immune serum from DSV4-immunized macaques was used in the FACS-based assay before depleting (un-depleted) or after depletion on immobilized MBP or one of the MBP-EDIII fusions.

*^b^*Values shown in each cell corresponds to virus neutralizing antibody titer (FNT_50_) measured against the DENV serotype indicated on top of the corresponding column.
